# Architecture-Level Event-Oriented Frontend for Heterogeneous Sensor Inference in Photonic Stochastic Systems

**DOI:** 10.3390/s26175565

**Published:** 2026-09-02

**Authors:** Oleg Angelsky, Myroslav Strynadko, Claudia Zenkova, Roman Zaiats, Xinzheng Zhang, Jun Zheng, Jingxian Cai

**Affiliations:** 1 Department of Correlation Optics, Yuriy Fedkovych Chernivtsi National University, Kotsiubynskoho Street, 58002 Chernivtsi, Ukraine; o.angelsky@chnu.edu.ua (O.A.); k.zenkova@chnu.edu.ua (C.Z.); zaiats.roman@chnu.edu.ua (R.Z.); 2 Optoelectronic Information Center, Taizhou Institute of Zhejiang University, Taizhou 318000, China; jingxiancai55@163.com; 3 The MOE Key Laboratory of Weak-Light Nonlinear Photonics, TEDA Institute of Applied Physics and School of Physics, Nankai University, Tianjin 300457, China; zxz@nankai.edu.cn

**Keywords:** heterogeneous sensors, stochastic computing, photonic stochastic systems, event-level inference, Bernoulli bitstreams, multisensor fusion, edge frontend, polarization encoding, synchronization, decorrelation

## Abstract

**Highlights:**

**What are the main findings?**

- An architecture-level event-oriented frontend was developed to convert heterogeneous sensor measurements into unified event-oriented probabilities and polarization-compatible Bernoulli optical bitstreams for photonic stochastic processing.
- Reliability-aware weighting, stochastic stream decorrelation, and time-varying synchronization control were identified as key design factors governing event-inference accuracy, robustness, and scaling behavior.
- The validation framework evaluates first-order photonic-interface nonidealities and demonstrates the frontend using a literature-grounded 520 nm optical smoke-response channel.

**What are the implications of the main findings?**

- The proposed architecture enables heterogeneous sensors to interface with photonic stochastic processors through a common event-level representation rather than through raw physical units.
- The reported design rules for weighting, decorrelation, synchronization, and robustness provide a practical basis for scalable multisensor edge inference in future photonic sensing systems.

**Abstract:**

Heterogeneous sensor systems generate measurements in incompatible physical units, which complicates their direct integration with photonic stochastic processors. This study proposes an architecture-level event-oriented frontend that converts heterogeneous sensor channels into unified event-oriented probabilities and then into Bernoulli bitstreams compatible with polarization-encoded optical interfaces. The framework combines sensor-to-probability mapping, weighted event-level fusion, stochastic bitstream generation, and system-level control of correlation and synchronization. Its performance was investigated through reproducible Colab-based modeling using baseline validation, weighting-strategy comparison, static and time-varying decorrelation/synchronization studies, and robustness/scaling analysis. The validation framework also includes a first-order analysis of photonic-interface nonidealities and a semi-real validation case using a digitized 520 nm optical smoke-transmittance response from the literature. The results show that the stochastic event estimate converges toward the float reference with increasing bitstream length, reliability-aware weighting outperforms equal and tested data-driven weighting in the benchmark, independent stream generation provides the best inference quality, and synchronization mismatch becomes measurable in time-varying fusion. The frontend also demonstrates graceful degradation under channel corruption and favorable scaling under mixed informative, weak, redundant, conflicting, and noisy channel configurations. These findings indicate that heterogeneous sensors can be interfaced with photonic stochastic systems through a common event-level representation and that weighting, decorrelation, synchronization, and robustness must be treated as core frontend design variables.

## 1. Introduction

Modern sensing systems increasingly rely on heterogeneous data sources, including environmental, optical, electrical, inertial, and chemical channels, whose outputs differ in physical units, dynamic ranges, sampling rates, and uncertainty profiles. Although such diversity can improve situational awareness and decision quality, it also makes direct joint processing difficult, especially when the target system is not a conventional digital processor but a stochastic or photonic computing platform. In parallel, multi-source information fusion has matured into a broad research field spanning probabilistic, evidence-based, and learning-based methods, while sensor-fusion applications continue to expand in localization, monitoring, and event-detection tasks [1,2,3,4]. However, heterogeneous sensor fusion is still most often formulated either in the native physical units of the sensors or after domain-specific feature engineering, which does not naturally provide a standardized interface for photonic stochastic processing.

Stochastic computing offers an appealing conceptual basis for such an interface because probabilities can be represented directly by bitstream statistics, enabling compact arithmetic primitives and tolerance to certain classes of noise and hardware imperfections [5]. Closely related probabilistic hardware concepts, such as p-bits and probabilistic logic, further emphasize the usefulness of tunable stochastic representations for inference-oriented architectures [6]. These ideas are attractive for edge sensing, where the primary requirement is often not exact reconstruction of a raw waveform but reliable estimation of an event, risk, or decision variable from multiple imperfect channels.

At the same time, photonic computing has advanced rapidly from conceptual optical accelerators to high-throughput experimental systems, including photonic convolutional processors and continuous-time tensor-core style architectures [7,8]. Reviews of analog optical computing and intelligent photonics also show that photonic platforms are increasingly being considered for real-time data processing because of their high bandwidth, parallelism, and energy-efficiency potential [9,10]. More recently, edge and in-sensor computing studies have argued that future sensing systems should not merely transmit raw measurements but should increasingly transform data into computation-ready forms near the sensing node itself [11]. Emerging photonic edge-intelligence demonstrations go even further by showing that multimodal analog inputs can be fused into optical representations suitable for low-latency processing [12].

Despite these advances, an important architectural gap remains. Existing sensor-fusion studies typically optimize algorithms for specific tasks and sensor combinations, whereas stochastic-computing studies focus on probabilistic representations and arithmetic, and photonic-computing studies focus on optical hardware acceleration. What is still insufficiently developed is a reusable architecture-level frontend that converts heterogeneous sensor channels into a common event-oriented stochastic interface that can be consumed by a photonic stochastic processor. In such a frontend, the goal is not to preserve a complete raw signal description or to reconstruct an original waveform, but to map each channel into a normalized contribution to an event of interest, expressed as a probability p_i_ ∈ [0, 1], and then to encode this contribution into a Bernoulli bitstream compatible with a physical optical interface. This distinction is important: the present work addresses event-level multisensor interfacing, not compact representation of a single signal waveform.

In this paper, we propose an architecture-level event-oriented frontend for heterogeneous sensors in photonic stochastic systems. The frontend maps heterogeneous measurements into a unified event-oriented probability space, generates Bernoulli bitstreams, and defines a polarization-compatible optical abstraction in which stochastic binary states can be carried by orthogonal optical states. On top of this representation, the system performs weighted multisensor fusion to estimate event probability. Beyond the architectural proposal itself, this study systematically investigates the roles of weighting strategy, stochastic stream decorrelation, synchronization, robustness under channel degradation, and scaling with increasing channel count. The computational validation shows that stochastic event estimates converge to float-reference inference with increasing bitstream length, that reliability-aware weighting is more effective than naive equal weighting in the tested benchmark, that independent bitstream generation outperforms shared-randomness policies, that synchronization mismatch becomes measurable in time-varying fusion, and that the proposed frontend degrades gracefully under missing, noisy, or perturbed channels while benefiting from structured scaling in mixed heterogeneous configurations. These results support the use of a unified event-level stochastic interface as a practical bridge between heterogeneous sensing and future photonic stochastic processors.

## 2. Related Work

Heterogeneous sensor fusion has been developed across probabilistic, evidence-based, and learning-based paradigms, with applications ranging from environmental monitoring and pedestrian detection to localization and industrial diagnostics. Recent work in Sensors and related journals shows that combining complementary sensing modalities can improve robustness and decision quality, especially when individual channels are noisy or incomplete. At the same time, these studies usually remain task-specific: they are designed for a fixed sensor set, a fixed inference target, and a conventional digital processing backend rather than for a reusable stochastic interface applicable across heterogeneous sensor classes [13,14,15,16].

Stochastic computing provides a different perspective by representing probabilities through bitstream statistics, enabling arithmetic and inference directly in the probability domain. Foundational survys established the core advantages and limitations of stochastic computing, including compact hardware realization, fault tolerance in certain regimes, and sensitivity to correlation between streams. More recent probabilistic hardware concepts, especially p-bit-based architectures, further reinforced the idea that controlled stochastic representations can support inference-oriented computation rather than exact deterministic reconstruction. However, most such works focus on representation, arithmetic primitives, logic, or hardware behavior, rather than on the problem of interfacing heterogeneous real-world sensors through a unified event-oriented frontend [1,2].

In parallel, photonic computing has advanced rapidly as a platform for high-bandwidth and parallel information processing. More broadly, recent optics and photonics reviews have also emphasized the relevance of coherence-related and structured optical-field concepts for practical optical diagnostics and advanced photonic applications [17]. Experimental demonstrations of photonic accelerators, optical convolutional processors, and tensor-style photonic computing architectures have shown that optical systems can implement computationally meaningful transforms at high throughput. Reviews of analog and intelligent photonics also emphasize the growing relevance of optical computing for artificial intelligence and low-latency signal processing. Still, these studies mainly address the computing substrate itself; they do not usually define how diverse sensor measurements should be normalized, weighted, synchronized, and stochastically encoded before entering a photonic processor [3,4].

Work on integrated optoelectronic sensing and emerging photodetector technologies further reinforces the need to treat the sensing-to-computation interface as a physical layer rather than only as an algorithmic mapping. Low-dimensional and two-dimensional material platforms are increasingly discussed as candidates for integrated sensing and in situ information encoding, while unified photodetector-evaluation guidelines emphasize that responsivity, dark current, noise, bandwidth, and device-level measurement conditions must be reported consistently when emerging optical sensing components are assessed [18,19]. These considerations support the view that photonic stochastic interfaces should be evaluated not only by abstract inference accuracy, but also by their sensitivity to optical-interface nonidealities such as loss, leakage, detector imbalance, and noise.

A related line of research comes from edge and in-sensor computing. These works argue that modern sensing systems should perform at least part of representation building and inference close to the sensing node, rather than forwarding only raw data to a remote processor. Recent photonic edge-intelligence studies extend this argument by showing that multimodal analog information can be processed in compact optical hardware. Nevertheless, even in these cases, the sensor-to-computation interface is typically application-specific. A general-purpose stochastic frontend for heterogeneous channels, especially one formulated around event-level probabilities and optical bitstream compatibility, remains insufficiently developed [9,10,11,16].

This gap is particularly important when the target task is not full waveform recovery but event-level inference. In many sensing scenarios, the relevant output is a probability of occurrence, risk, anomaly, or decision state rather than a faithful reconstruction of each raw signal. From that viewpoint, heterogeneous sensor channels do not need to be fused in their native physical units; instead, they can be mapped into normalized event contributions and then processed in a common stochastic domain. This shift from raw-signal fusion to event-oriented interfacing is central to the present study [13,14,15].

The novelty of the present work is therefore not the isolated use of probability mapping or weighted fusion, which are well known in probabilistic sensor fusion. Rather, the contribution lies in treating these operations as part of a reusable sensor-to-stochastic frontend for photonic stochastic systems. Conventional sensor-fusion pipelines usually optimize a task-specific digital inference model for a fixed set of sensors. Stochastic-computing studies usually focus on arithmetic primitives, bitstream representations, or probabilistic logic. Photonic-computing studies usually focus on the optical processing substrate. In contrast, the present work addresses the intermediate architectural layer: how heterogeneous physical sensor outputs can be mapped, weighted, decorrelated, synchronized, and encoded into Bernoulli streams suitable for a polarization-compatible optical stochastic interface. This frontend-level focus distinguishes the proposed architecture from both conventional probabilistic fusion and photonic accelerator studies.

## 3. System Architecture

**Figure 1:**
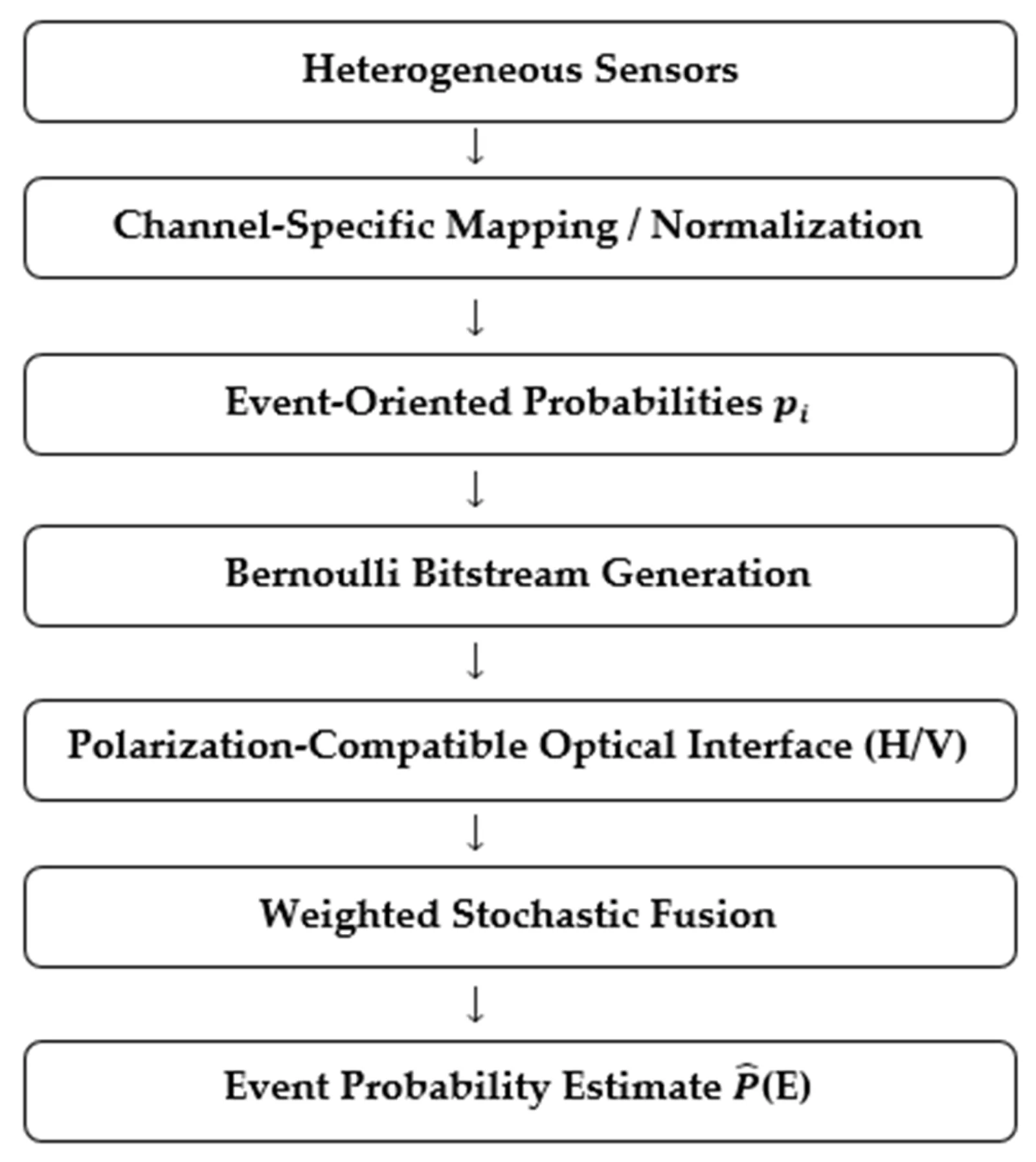
Block diagram of the proposed event-oriented edge stochastic frontend for heterogeneous sensors and its polarization-compatible photonic interface.

The proposed system is organized as an architecture-level event-oriented frontend that converts heterogeneous sensor measurements into a common stochastic representation suitable for event-level inference in photonic stochastic systems. The overall architecture of the proposed event-oriented stochastic frontend is shown in Figure 1.

Heterogeneous sensor measurements are first converted into channel-specific event-oriented probabilities $p_{i}\in [0,\;1]$, then encoded into Bernoulli bitstreams, and finally delivered to a stochastic fusion block through a polarization-compatible optical abstraction in which orthogonal optical states represent stochastic binary states.

The architecture is designed to decouple the physical diversity of primary sensors from the probabilistic format required by the downstream processor. Instead of forwarding raw measurements in their native units, the frontend transforms each channel into a normalized event-oriented contribution, encodes this contribution into a Bernoulli bitstream, and provides an optical-compatible binary abstraction for subsequent stochastic fusion.

### 3.1. Heterogeneous Sensor Layer

The input layer may include channels of different physical nature, such as temperature, humidity, wind, rainfall, optical smoke or aerosol sensing, gas concentration, electrical measurements, or other application-specific signals. In the benchmark used below, the optical smoke or aerosol channel is denoted as smoke_optical and represents an optical response associated with smoke-induced attenuation, obscuration, scattering, or photodiode-like detection. These channels generally differ in scale, direction of influence, noise characteristics, and temporal behavior. Therefore, they are not directly comparable at the processor interface. In the proposed architecture, each channel is treated first as a source of evidence about a target event rather than as an isolated physical signal. This distinction allows the frontend to operate on heterogeneous sensing systems without requiring a common physical unit across channels.

### 3.2. Event-Oriented Mapping

For each channel *x*_*i*_, the frontend applies a channel-specific mapping function that produces a normalized event-related probability-like quantity *p*_*i*_ ∈ [0, 1]:

*p*_*i*_ = *g*_*i*_(*x*_*i*_), *p*_*i*_ ∈ [0, 1]. (1)

Here, *g*_*i*_(⋅) is not merely a numerical normalization operator; it expresses the contribution of channel *i* to the event of interest. Depending on the sensor semantics, the mapping may be direct or inverse. For a direct channel, larger measured values correspond to larger event contributions. For an inverse channel, larger measured values correspond to smaller event contributions. This formulation enables physically different inputs to be represented in a unified event-oriented space without forcing them into artificial equivalence in their native units.

**Figure 2:**
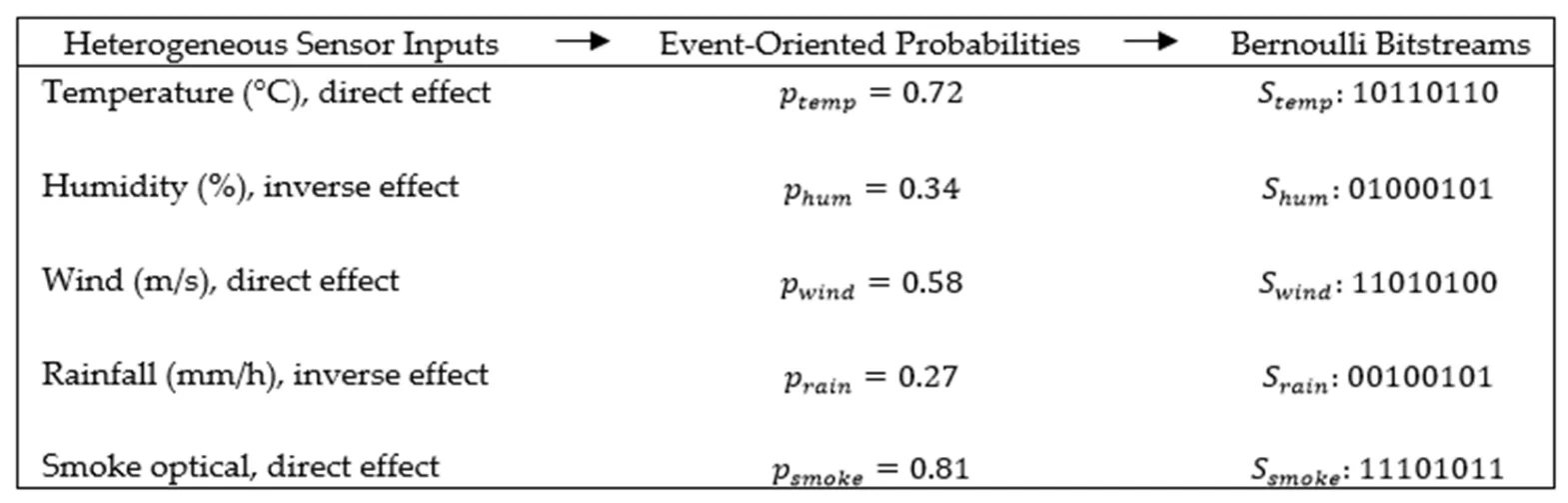
Transformation of heterogeneous sensor inputs into a unified stochastic event representation. The arrows indicate the processing sequence from raw heterogeneous sensor inputs to event-oriented probabilities and then to Bernoulli bitstreams. Physically different sensor channels with direct or inverse event influence are mapped into normalized event-oriented probabilities and then converted into Bernoulli bitstreams, enabling multisensor fusion in a common stochastic domain.

Figure 2 illustrates how heterogeneous sensor inputs are transformed into event-oriented probabilities and then into Bernoulli bitstreams compatible with the optical interface.

Physically different sensor channels with direct or inverse event influence are mapped into normalized event-oriented probabilities and then converted into Bernoulli bitstreams, enabling multisensor fusion in a common stochastic domain.

In practice, *g*_*i*_(⋅) may be implemented by linear normalization, thresholded mapping, bounded nonlinear transformation, or a feature-based local preprocessing block. Importantly, such feature extraction is treated here as an auxiliary frontend mechanism, not as the main contribution of the work.

### 3.3. Stochastic Edge Representation

After event-oriented mapping, each channel probability *p*_*i*_ is converted into a Bernoulli bitstream:

*Si* ∼ Bernoulli(*pi*). (2)

In the static formulation, the bitstream encodes the average event contribution of the channel over an observation window. In the time-varying formulation, the frontend produces slot-wise stochastic representations:

*S*_*i*_(*t*) ∼ Bernoulli(*p*_*i*_(*t*)), (3)

where *t* denotes the time slot. This second formulation is especially important when the target event evolves in time and when synchronization between channels must be explicitly controlled.

The use of Bernoulli streams serves two roles. First, it creates a common stochastic protocol for heterogeneous sensor channels. Second, it makes the frontend compatible with stochastic computing and photonic binary-state implementations, where information is naturally processed in probabilistic bitstream form.

### 3.4. Polarization-Compatible Optical Interface

To bridge the edge frontend and a photonic stochastic processor, the proposed architecture uses a polarization-compatible optical abstraction in which the two logical stochastic states are represented by orthogonal optical states. In the simplest formulation, the Bernoulli states are associated with orthogonal polarization components: *H* → 1, *V* → 0.

This representation is not introduced as a complete hardware realization in the present paper, but as a physically meaningful interface layer between edge-generated stochastic bitstreams and downstream photonic processing blocks, which is consistent with the broader trend toward photonic architectures for high-throughput and parallel information processing [3,4]. Such an abstraction is useful because it provides a direct correspondence between stochastic binary coding and optical state encoding, which is compatible with polarization-based photonic logic and measurement schemes.

In a physical photonic implementation, the polarization-compatible interface represents a binary-state optical layer with finite transmission, finite extinction ratio, detection accuracy limits, and noise tolerance. The nominal H- and V-associated states may therefore be affected by differential loss, polarization leakage, detector gain imbalance, offsets, and additive noise. These perturbations first reduce the analog separation between the two optical-state representations and may lead to hard bit errors only under stronger degradation. An implementation-level scheme and a first-order nonideality analysis of this interface are provided in Supplement S5 of the Supplementary Material. A practical implementation path can be defined at the component level as follows. The event-oriented probabilities are first converted into electronic Bernoulli control streams by the edge frontend. These streams can then drive an optical binary-state encoder, for example, through an electro-optic intensity or polarization modulator, a polarization switch, or a polarization-selective routing stage. A laser or LED source provides the optical carrier, while the logical stochastic states are encoded as nominal H- and V-associated optical states. The encoded stream can then be routed through polarization-maintaining waveguides or bulk-optical polarization components, analyzed by polarization-selective elements such as PBS/analyzer stages, and detected by photodiodes or balanced detector pairs before event-level readout. In such a device path, the key parameters requiring calibration would include modulator extinction ratio and bandwidth, insertion and differential loss, polarization extinction ratio, crosstalk, detector responsivity, dark current, gain mismatch, offsets, additive noise, timing jitter, and thermal drift. The present study treats this path at the architecture and first-order interface level, while full optimization of a specific material platform, photonic layout, and detector structure remains a subject for future hardware-oriented work.

### 3.5. Event-Level Fusion Block

The downstream processor operates not on raw sensor values, but on the normalized stochastic contributions of all channels. In the float reference model, multisensor fusion is performed by a weighted aggregation:

(4) $$z=\sum_{i=1}^{M}w_{i}p_{i},$$

followed by a nonlinear decision mapping:

(5) P(E)=σ(α(z−θ)),

where *w*_*i*_ are channel weights, *θ* is the decision threshold, *α* is a slope parameter, and *σ*(⋅) is the sigmoid function. In the stochastic domain, the same logic is applied to empirical bitstream-derived estimates $\hat{p}$_*i*_ or $\hat{p}$_*i*_(*t*), producing an estimated event probability $\hat{P}$(*E*) or $\hat{P}$(*E*,*t*).

This architecture, therefore, treats event inference as a probabilistic fusion problem in a common stochastic representation space. The frontend is reusable in the sense that heterogeneous sensing modalities are converted into the same interface format before fusion, while the application-specific semantics are captured through the mapping rules and weights.

### 3.6. Core Design Variables

A key feature of the proposed architecture is that inference quality depends not only on the mapping *x*_*i*_ → *p*_*i*_ but also on several system-level design variables:

- Weight assignment. The influence of each sensor channel is controlled by *w*_*i*_, which may be assigned equally, manually, reliability-aware, or by data-driven calibration.
- Bitstream precision. The length of the Bernoulli stream determines the stochastic estimation accuracy and convergence to the float reference.
- Correlation policy. Independent, grouped, or shared-randomness stream generation changes the effective inter-channel correlation and therefore the fusion quality.
- Synchronization mode. In time-varying inference, perfect alignment, fixed lag, or random jitter affect the temporal consistency of multisensor fusion.
- Robustness conditions. Noise, channel dropout, missing slots, and weight perturbations influence both average inference error and temporal event tracking quality.

These variables are not secondary implementation details; they are integral parts of the frontend architecture. Accordingly, the remainder of this paper evaluates the proposed system not only as a conceptual interface but also as a design space with measurable effects on inference accuracy, robustness, and scaling.

### 3.7. Architectural Scope of the Present Study

The present work focuses on the architectural and computational validation of the frontend. It demonstrates how heterogeneous sensor channels can be transformed into a unified stochastic event-level interface and how this interface behaves under different fusion, synchronization, decorrelation, robustness, and scaling conditions. Detailed reproducible studies supporting these architectural claims are provided in the Supplementary Material: baseline frontend validation in Supplement S1, weighting analysis in Supplement S2, decorrelation analysis in Supplement S3, time-varying synchronization analysis in Supplement S3b, robustness/scaling analysis in Supplement S4, first-order photonic-interface nonideality analysis in Supplement S5, and semi-real literature-grounded optical validation in Supplement S6.

This study is positioned as an architecture-level frontend model rather than as a complete photonic hardware prototype. Within this scope, the Supplementary Material provides two complementary validation layers. Supplement S5 analyzes a physically interpretable first-order optical-interface layer, including differential loss, leakage, detector imbalance, offsets, and noise. Supplement S6 complements the controlled benchmark with a literature-grounded optical smoke-response validation case based on a digitized 520 nm transmittance curve. Together, these analyses support the practical interpretation of the proposed frontend while preserving its primary focus on event-oriented stochastic interfacing.

## 4. Methods

This section formalizes the proposed architecture-level event-oriented frontend and the computational framework used for its validation. The method is organized around five stages: event-oriented channel mapping, float-reference fusion, stochastic bitstream generation, correlation/synchronization control, and robustness/scaling evaluation. The formulation is intentionally architecture-oriented: its purpose is not to reconstruct original waveforms, but to provide a common event-level stochastic interface for heterogeneous sensors.

### 4.1. Event-Oriented Sensor Mapping

Let $x_{i}\;$ denote the measurement of sensor channel i, where i = 1, …, *M*. Because channels may differ in units, ranges, and semantics, each channel is converted into a normalized event-oriented quantity (1). For direct channels, larger values of *x*_*i*_ correspond to larger event contributions. For inverse channels, larger values correspond to smaller event contributions. In the simplest bounded linear form,

(6) $${\tilde{x}}_{i}=\frac{x_{i}-x_{i,min}}{x_{i,max}-x_{i,min}},$$

with clipping to [0, 1], and

(7) $${\tilde{x}}_{i}\;,\;\;\;p_{i}=\{\begin{array}{ll} {\tilde{x}}_{i}, & for\;direct\;channels,\;\;\\ 1-{\tilde{x}}_{i}, & for\;inverse\;channels \end{array}$$

The parameters $x_{i,min}$ and $x_{i,max}$ define the operating range used for channel i. They may be chosen from sensor specifications, calibration measurements, historical operating data, or application-specific safety thresholds. The mapping does not imply that temperature, humidity, wind, rainfall, and optical smoke response become physically identical quantities. Instead, each channel is converted into a dimensionless event-support variable whose direction is determined by the event semantics. Thus, a direct channel contributes more strongly as its measured value increases, whereas an inverse channel contributes less strongly as its measured value increases.

In practical use, the mapping functions $g_{i}$(⋅) should be treated as calibrated frontend components. Their parameters may be obtained from sensor specifications, calibration measurements, expert-defined operating thresholds, historical data, or task-specific validation datasets. The simple bounded linear mapping used in the benchmark is therefore one transparent implementation of the general event-oriented mapping principle. More complex applications may replace it with a sigmoid, thresholded, piecewise, or feature-based mapping function, provided that the resulting output remains a bounded event-support variable $p_{i}\in$ [0, 1]. The reproducibility of this frontend layer depends on explicitly reporting the mapping ranges, directions, and calibration assumptions for each channel.

### 4.2. Float-Reference Event Fusion

The deterministic reference event score is defined as a weighted combination of the mapped channel probabilities (4), where $w_{i}\;\geq \;0$ are normalized channel weights satisfying

(8) $$\sum_{i=1}^{M}w_{i}=1.$$

The corresponding float event probability is then computed using a sigmoid decision model (5), where *σ*(*u*) = 1/(1 + *e*^−*u*^).

This float model serves as the reference against which stochastic inference is compared. It is not introduced as the only possible fusion law, but as a transparent and reproducible event-level benchmark.

### 4.3. Bernoulli Bitstream Representation

Each normalized channel contribution $p_{i}\;$ is converted into a Bernoulli bitstream (2). For a bitstream of length N, the empirical channel estimate is

(9) $${\hat{p}}_{i}=\frac{1}{N}\sum_{k=1}^{N}S_{i,k}$$

The stochastic event estimate in the static setting is then

(10) $$\hat{P}(E)=\sigma \left(\alpha \left(\sum \sum_{i=1}^{M}w_{i}{\hat{p}}_{i}-\theta \right)\right).$$

This formulation allows direct evaluation of the convergence of stochastic inference toward the float reference as the bitstream length increases, consistent with the standard stochastic-computing interpretation of probability through bitstream statistics [1].

### 4.4. Time-Varying Slot-Wise Formulation

To study synchronization effects, a time-varying formulation is introduced. In this case, each channel is represented by a probability trajectory $p_{i}(t)$, where t = 1, …, *T* denotes the time slot. The slot-wise Bernoulli representation is (3). For each slot, a local bitstream of length $N_{bits}$ is generated, yielding

(11) $${\hat{p}}_{i}\left(t\right)=\frac{1}{N_{bits}}\sum_{k=1}^{N_{bits}}S_{i,k}\left(t\right).$$

The time-varying stochastic event estimate becomes

(12) $$\hat{P}(E,t)=\sigma \left(\alpha \left(\sum \sum_{i=1}^{M}w_{i}{\hat{p}}_{i}\left(t\right)-\theta \right)\right).$$

This formulation is crucial because synchronization mismatch is largely invisible if inference is based only on full-stream averages. In the slot-wise model, temporal misalignment between channels can directly distort event tracking.

### 4.5. Weight Assignment Strategies

Four weighting strategies were examined in the computational benchmark. First, equal weighting assigns $w_{i}=1/M$, treating all channels identically. Second, expert weighting uses manually chosen weights derived from assumed channel relevance. Third, reliability-aware weighting combines expert importance with a channel reliability factor $r_{i}$, so that $w_{i}\propto \alpha_{i}r_{i}$, followed by normalization to unit sum.

Fourth, a tested data-driven weighting variant estimates weights from synthetic event labels using logistic regression. In the benchmark, this calibration used the same controlled five-channel scenario and served as a simple supervised baseline rather than as an optimized machine-learning fusion model. The estimated coefficients were projected to nonnegative values and normalized to a unit sum to preserve the same interpretable weighted-fusion structure used by the other strategies. This procedure allows a controlled comparison between manually specified, reliability-aware, and simple data-driven weighting, but it should not be interpreted as an exhaustive evaluation of supervised fusion methods. More advanced Bayesian, neural, or context-adaptive weighting approaches may outperform this simple calibration strategy in other datasets and are outside the scope of the present frontend-focused study.

These strategies are compared in terms of inference accuracy and threshold consistency.

### 4.6. Correlation Policies for Stochastic Streams

Because stochastic fusion is sensitive to inter-stream correlation, three stream-generation policies were considered [1].

In the independent policy, each channel uses an independent source of randomness.

In the shared policy, all channels use a common random sequence, creating strong inter-channel correlation.

In the grouped policy, subsets of channels share random sequences within predefined groups, while the remaining channels remain independent. This produces partial correlation and intermediate behavior between the fully independent and fully shared cases.

These policies are used to evaluate how correlation structure affects multisensor event inference.

### 4.7. Synchronization Policies

Two synchronization formulations were studied.

In the static formulation, synchronization mismatch was introduced as circular shifts within generated bitstreams. This provided a preliminary check, but such shifts do not substantially alter empirical means and therefore only weakly affect full-stream inference.

In the time-varying formulation, synchronization mismatch was applied across the time-slot axis, making it directly relevant for slot-wise event tracking. The following modes were considered:

perfect_sync: no temporal shift,

fixed_lag_1, fixed_lag_4, fixed_lag_8: selected channels shifted by a fixed number of slots,

random_jitter_2, random_jitter_6: channel-wise random shifts within bounded lag intervals.

This second formulation allows synchronization errors to be quantified as a true system-level factor.

### 4.8. Robustness Scenarios

Robustness was evaluated using four classes of perturbations.

First, additive channel noise was injected into selected channels at increasing noise levels.

Second, single-channel dropout was implemented by replacing one channel at a time with a neutral value, thereby estimating channel criticality.

Third, missing-slot corruption was introduced by replacing a fraction of time slots in selected channels with neutral values.

Fourth, weight perturbation was studied using both global random perturbation and targeted modification of dominant or weak channels.

Together, these scenarios test whether the frontend exhibits graceful degradation under realistic forms of degradation or misconfiguration.

### 4.9. First-Order Photonic-Interface Nonideality Analysis

The polarization-compatible interface is represented as a first-order optical binary-state layer in Supplement S5 of the Supplementary Material. This layer is not intended as a full model of a complete photonic chip or a specific fabricated device; rather, it provides a controlled perturbation model for the H/V optical binary-state interface. The considered nonidealities include differential transmission between the H- and V-associated branches, polarization leakage between nominally orthogonal states, detector gain imbalance, detector offsets, and additive detection noise.

The first-order interface model is formulated at the level of optical-state transmission, leakage, detection imbalance, offsets, and noise. It does not resolve microscopic material-transport processes inside a specific photonic device. Effects associated with phonons, carrier–phonon interactions, thermo-optic response, material heating, or detector-noise mechanisms are therefore represented only indirectly through the selected interface-level perturbation parameters. A more detailed treatment of these effects would require a defined material platform, device geometry, modulation mechanism, and detector structure, and is therefore more appropriate for future hardware-specific modeling.

The interface response is characterized using both hard-domain and soft-domain diagnostics. Hard-domain behavior is evaluated through the recovered binary states and bit error rate, whereas soft-domain behavior is evaluated through the analog ratio variable and the resulting event-level error. This distinction is important because optical degradation can first reduce analog state separation before producing observable hard bit errors. Accordingly, MAE_soft, QSeparation, and BER are interpreted jointly as complementary indicators of photonic-interface degradation rather than treating BER as the only performance metric.

### 4.10. Semi-Real/Literature-Grounded Validation

Supplement S6 of the Supplementary Material provides a semi-real literature-grounded validation layer based on an externally measured optical smoke-response proxy. The optical channel is derived from a digitized 520 nm smoke-layer transmittance curve reported in a published multi-wavelength densitometer study [20]. The digitized transmittance signal is converted into optical obscuration and then normalized as an event-oriented smoke-probability channel. This externally grounded optical channel is combined with controlled semi-real environmental proxy channels and processed through the same event-oriented mapping, Bernoulli bitstream generation, and float-versus-stochastic inference pipeline used in the main benchmark.

This validation layer is intended as an externally grounded extension of the controlled benchmark rather than as a new independent field experiment. In this context, the term semi-real means that the optical smoke channel is derived from external published experimental data, whereas the remaining environmental channels are controlled proxy trajectories aligned to the same event timeline. It evaluates whether the proposed frontend preserves its event-oriented stochastic inference behavior when one of the input channels is derived from experimentally reported optical sensing data.

### 4.11. Scaling Configurations

To study scaling, the frontend was evaluated with systems of increasing size: 3, 5, 8, 12, and 16 channels.

The added channels were not restricted to uniformly informative channels. Instead, mixed sets were constructed that included informative, weak, redundant, conflicting, and noisy channels. This makes the scaling study more representative of practical heterogeneous sensor systems, where additional channels are not always equally beneficial.

### 4.12. Time-Varying Benchmark Construction

For the time-varying studies, synthetic channel probability trajectories were generated using a combination of: baseline channel levels, slow temporal modulation, random fluctuations, and localized event-related pulses.

This yielded structured multisensor trajectories in which the event probability evolves over time rather than remaining static. The benchmark was designed not as a domain-specific physical simulator, but as a reproducible architecture-level testbed for studying frontend behavior under controlled heterogeneous conditions.

### 4.13. Evaluation Metrics

The following metrics were used throughout this study.

The mean absolute error:

(13) $$MAE=\frac{1}{K}\sum_{j=1}^{K}|y_{j}-{\hat{y}}_{j}|,$$

and the root mean square error:

(14) $$RMSE=\sqrt{\frac{1}{K}\sum_{j=1}^{K}{\left(y_{j}-{\hat{y}}_{j}\right)}^{2}},$$

where $y_{j}$ and ${\hat{y}}_{j}$ denote reference and estimated values.

Bias was defined as

(15) $$Bias=\frac{1}{K}\sum_{j=1}^{K}\left(y_{j}-{\hat{y}}_{j}\right),$$

For event decisions, threshold agreement was computed as the fraction of samples or time slots for which the binarized reference and estimated event decisions matched.

For time-varying inference, temporal consistency was additionally quantified by the peak alignment error, defined as the mean absolute difference between the slot index of the reference event peak and the slot index of the estimated event peak.

### 4.14. Reproducibility and Supplementary Implementation

All computational studies were implemented in reproducible Colab notebooks and organized into the Supplementary Materials. The final verification runs of the computational notebooks were executed in Google Colab using Python 3.12.13. The main software libraries used for numerical simulation, data processing, plotting, and calibration were NumPy 2.0.2, pandas 2.2.2, Matplotlib 3.10.0, and scikit-learn 1.6.1. The package includes baseline frontend validation (Supplement S1), weighting strategies (Supplement S2), decorrelation study (Supplement S3), time-varying synchronization study (Supplement S3b), robustness/scaling study (Supplement S4), first-order photonic-interface nonideality analysis (Supplement S5), and semi-real literature-grounded validation (Supplement S6). These supplementary modules provide implementation details, plots, tables, data exports, and reproducibility artifacts supporting the main results of the paper.

## 5. Experimental Design

The experimental design was constructed to validate the proposed frontend at progressively higher levels of architectural complexity. Rather than relying on a single benchmark, this study was organized into a sequence of computational stages that examined baseline interface validity, weight assignment, stochastic correlation, synchronization, robustness, and scaling. This staged design allowed individual system factors to be isolated and then integrated into a broader evaluation of multisensor event inference.

### 5.1. Baseline Five-Channel Heterogeneous Benchmark

The core benchmark used throughout this study was a five-channel heterogeneous sensor set composed of temperature, humidity, wind, rainfall, and smoke_optical. The purpose of this benchmark is not to reproduce a complete domain-specific fire or environmental monitoring system, but to provide a controlled architecture-level testbed containing channels with different physical units, event semantics, temporal behavior, and inferred relevance.

The environmental channels represent typical variables that may contribute to fire-risk, smoke-related, or environmental event inference. Temperature and wind are treated as direct channels because larger values increase the event-support contribution in the tested benchmark. Humidity and rainfall are treated as inverse channels because larger values reduce the event-support contribution. The smoke_optical channel represents an optical smoke or aerosol response, such as attenuation, obscuration, scattering, or a photodiode-like signal caused by smoke particles in an optical path. It is therefore treated as a direct event-support channel.

In the controlled baseline benchmark, all five channels are generated synthetically in order to isolate the frontend behavior under reproducible conditions. Each raw channel is mapped into an event-oriented probability $p_{i}\in [0,\;1]$, encoded into Bernoulli bitstreams, and fused into an estimated event probability. The corresponding channel ranges, mapping directions, and weights are reported in the Supplementary Material, where the same five-channel configuration is used as the reference setup for Supplements S1–S4.

The smoke_optical abstraction is further connected to externally grounded optical sensing behavior in Supplement S6 of the Supplementary Material, where a digitized 520 nm smoke-layer transmittance curve is converted into an event-oriented optical smoke probability. Thus, the baseline smoke_optical channel should be understood as a controlled optical-smoke response variable in the synthetic benchmark and as a bridge to the literature-grounded optical validation layer.

### 5.2. Baseline Validation Stage

The first experimental stage was designed to validate the frontend itself. In this stage, heterogeneous raw channels were generated synthetically, mapped into event-oriented probabilities, and used to compute both a float reference event probability and its stochastic estimate. The principal variable in this stage was the Bernoulli bitstream length N, which was swept across increasing values to evaluate convergence.

The objective of this stage was to answer the most basic architectural question: does the proposed frontend preserve event-level inference quality when heterogeneous channel contributions are encoded stochastically? Detailed implementation and convergence results are provided in Supplement S1 of the Supplementary Materials.

### 5.3. Weighting-Strategy Comparison

The second stage examined weight assignment as a design variable rather than as a fixed constant. Using the same baseline benchmark, four weighting strategies were compared: equal weighting, expert weighting, reliability-aware weighting, and data-driven weighting.

For the data-driven case, synthetic event labels were generated from a hidden reference rule, allowing weights to be estimated through logistic-regression-based calibration. The resulting stochastic event estimates were compared against both their own float baselines and a common reference event model.

This stage was designed to determine whether weight assignment materially changes multisensor fusion quality and whether a reliability-aware strategy offers practical advantages over naive equal weighting or the tested data-driven calibration variant. Detailed results are provided in Supplement S2 of the Supplementary Materials.

### 5.4. Static Correlation Study

The third stage investigated the effect of the correlation between stochastic bitstreams. Three stream-generation policies were compared: independent, shared, and grouped.

In the independent case, each sensor channel was assigned its own randomness source. In the shared case, all channels used a common randomness source. In the grouped case, subsets of channels shared randomness while others remained independent. These policies were tested first in a static setting using full-stream probability estimation.

This stage was introduced to isolate the influence of stochastic correlation structure on multisensor event inference. Because stochastic arithmetic is known to be sensitive to correlation, the objective here was to establish decorrelation as a system-level design requirement. Detailed results are provided in Supplement S3 of the Supplementary Materials.

### 5.5. Time-Varying Synchronization Study

The static correlation study was followed by a time-varying extension in which each channel was represented by a slot-wise probability trajectory $p_{i}(t)$. This second synchronization stage was necessary because simple circular shifts in static full-stream averaging do not strongly affect empirical bitstream means. To expose synchronization effects explicitly, the event estimate was reformulated as a time-resolved quantity $\hat{P}(E,t)$.

The following synchronization modes were then evaluated: perfect synchronization, fixed temporal lags, and random temporal jitter.

These were combined with the same three correlation policies: independent, shared, and grouped. This stage was intended to determine whether synchronization mismatch degrades temporal event tracking when multisensor fusion is performed slot-wise rather than through full-stream averaging. The corresponding reproducible implementation is provided in Supplement S3b of the Supplementary Materials.

### 5.6. Robustness Study

The robustness stage evaluated how the frontend behaves under common forms of degradation or misconfiguration. Four robustness categories were examined.

First, additive noise was injected into selected channels at progressively increasing levels.

Second, single-channel dropout was simulated by replacing one channel at a time with a neutral value, allowing channel criticality to be estimated.

Third, random missing-slot corruption was introduced in selected channels, with varying fractions of missing time slots.

Fourth, the assigned fusion weights were perturbed either globally or in a targeted manner, with separate attention to dominant and weak channels.

The purpose of this stage was to determine whether the frontend degrades gracefully and whether different channels contribute equally to the stability of event inference. The complete robustness analyses are given in Supplement S4 of the Supplementary Materials.

### 5.7. Scaling Study

The final experimental stage examined the effect of increasing the number of heterogeneous channels. Systems of 3, 5, 8, 12, and 16 channels were constructed. The added channels were intentionally heterogeneous not only in modality but also in informational value. Thus, the larger channel sets included not only informative channels but also weak, redundant, conflicting, and noisy channels.

This design was important because it reflects realistic scaling conditions more faithfully than a purely optimistic expansion with only useful channels. The objective was to determine whether performance continues to improve as the frontend grows and under what conditions mixed-quality channel scaling remains beneficial. These results are also reported in Supplement S4 of the Supplementary Materials.

### 5.8. Evaluation Protocol

Across all stages, the reference event model was computed in the float domain from the mapped channel probabilities and the selected weight configuration. Stochastic event estimates were then generated from Bernoulli bitstreams under the relevant generation, synchronization, robustness, or scaling conditions. The resulting reference and estimated event outputs were compared using: mean absolute error, root mean square error, bias, threshold agreement, and peak alignment error in time-varying scenarios.

The use of several complementary metrics was intentional. Average amplitude error alone may understate degradation when threshold decisions or temporal peak localization are affected. Therefore, the experimental design emphasized joint interpretation of amplitude, decision, and temporal-alignment quality.

### 5.9. Role of the Supplementary Materials in the Experimental Design

Because the present article focuses on architectural interpretation rather than code-level exposition, the experimental workflow was distributed between the main text and a structured supplementary package. The main paper reports the principal scenarios and findings, whereas the Supplementary Material provides the detailed notebook implementations, intermediate plots, parameter sweeps, and reproducibility files. Specifically: Supplement S1 supports baseline validation, Supplement S2 supports weighting analysis, Supplement S3 supports static decorrelation analysis, Supplement S3b supports time-varying synchronization analysis, Supplement S4 supports robustness and scaling analysis, Supplement S5 supports first-order photonic-interface nonideality analysis, and Supplement S6 supports semi-real literature-grounded optical validation.

This structure ensures that the article remains concise while still providing full computational traceability of the reported results.

## 6. Results

### 6.1. Baseline Validation of the Event-Oriented Frontend

The first result of this study is that the proposed frontend successfully converts heterogeneous sensor channels into a common event-oriented stochastic representation without losing the ability to reproduce the float-reference event estimate. In the baseline five-channel benchmark, the raw sensor channels exhibited clearly different statistical ranges and distributions, confirming that the input layer was genuinely heterogeneous rather than artificially homogenized. After channel-specific mapping, however, all channels were represented in the same normalized event-oriented space *pi* ∈ [0, 1], making them directly compatible with the stochastic fusion stage. Detailed distributions and mapping plots are provided in Supplement S1 of the Supplementary Materials.

**Figure 3:**
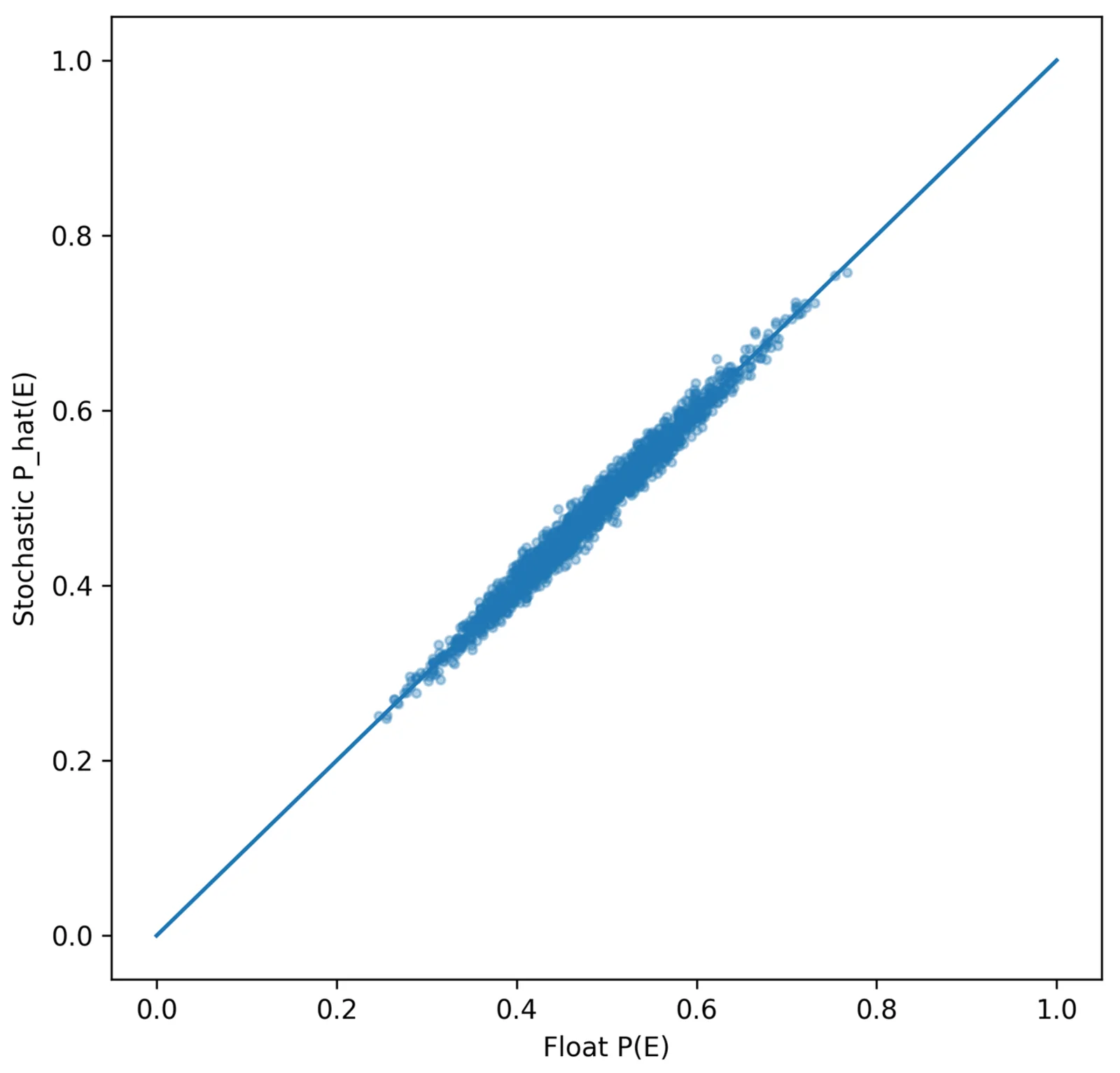
Comparison between the float reference event probability and the stochastic event estimate in the baseline heterogeneous benchmark at *N* = 256.

The float-versus-stochastic comparison also showed that even moderate stream lengths already provide useful inference fidelity. At N = 256, the stochastic event estimate was closely aligned with the float baseline, indicating that the interface does not require unrealistically long streams to achieve meaningful event-level agreement. The agreement between the float event model and the stochastic estimate is illustrated in Figure 3.

The figure illustrates the agreement between deterministic multisensor fusion and its stochastic approximation after channel mapping and Bernoulli bitstream generation.

This is important for practical edge implementations, where latency and bitstream length are constrained resources.

**Figure 4:**
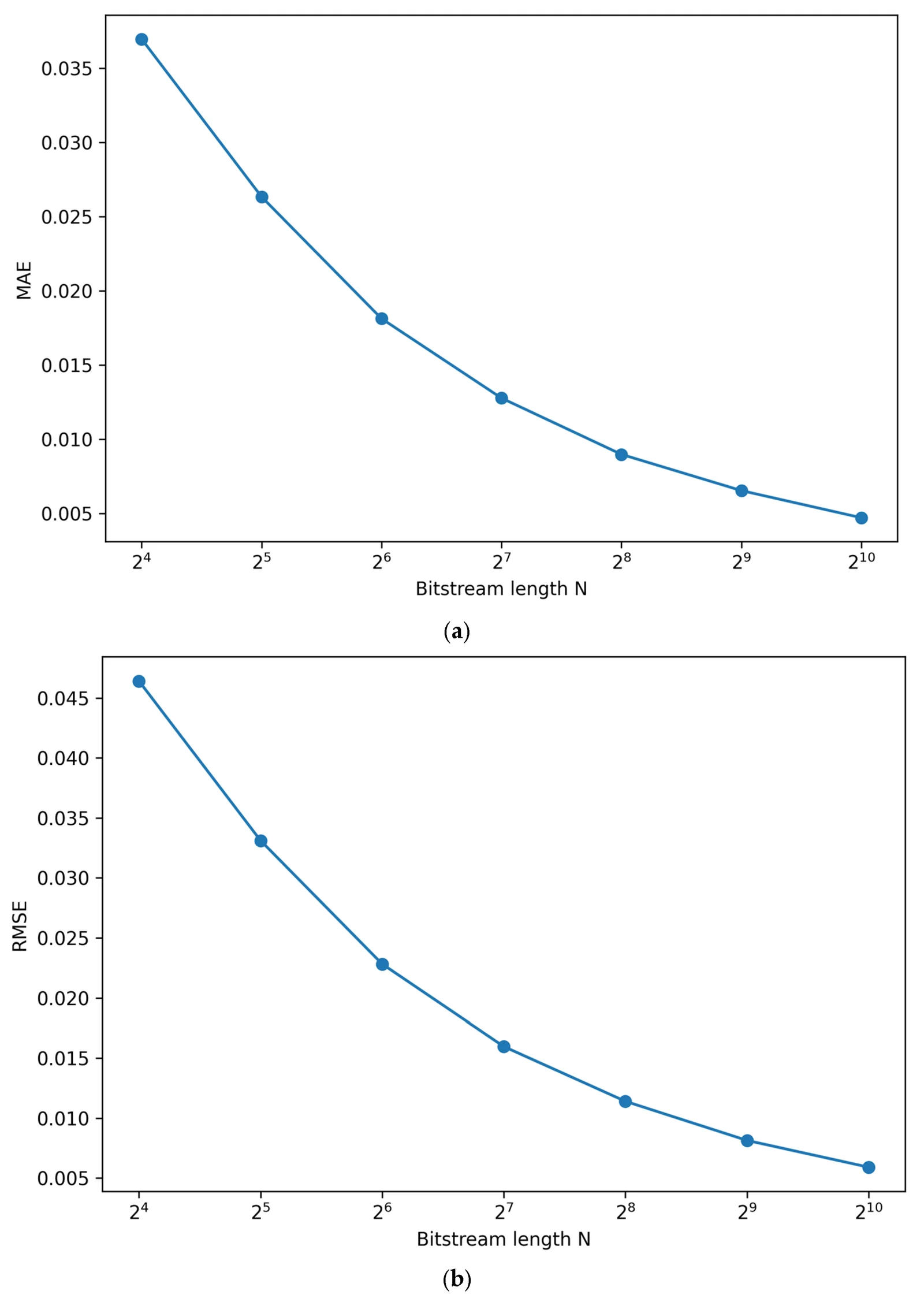
Convergence of stochastic event inference with increasing bitstream length. (**a**) Mean absolute error (MAE) versus bitstream length. (**b**) Root mean square error (RMSE) versus bitstream length.

The stochastic event estimate $\hat{P}(E)$ converged toward the float reference P(E) as the Bernoulli bitstream length increased. The convergence of stochastic inference with increasing bitstream length is summarized in Figure 4.

In the baseline validation, the mean absolute error decreased from 0.036966 at N = 16 to 0.008969 at N = 256, and further to 0.004697 at N = 1024. The corresponding RMSE decreased from 0.046415 at N = 16 to 0.011401 at N = 256, and to 0.005907 at N = 1024. These results confirm that the frontend supports controlled approximation of float-domain multisensor inference through stochastic encoding.

The approximately monotonic decrease of both MAE and RMSE with increasing N reflects the expected reduction of Bernoulli sampling uncertainty as the bitstream length increases. The improvement is strongest between N=16 and N=256, where stochastic sampling noise is still a dominant error source. Beyond this range, the curves flatten, indicating diminishing returns from further increasing bitstream length. This behavior is practically important because it suggests that moderate stream lengths can already provide useful event-level accuracy, while very long streams may not be necessary for all edge-inference settings.

Taken together, these results show that the proposed frontend satisfies its most basic requirement: heterogeneous channels can be transformed into a common stochastic interface, and the resulting event estimate converges reliably to the deterministic reference with increasing stochastic precision. This baseline result establishes the frontend as a valid architectural bridge between heterogeneous sensing and stochastic fusion. Additional convergence plots and implementation details are given in Supplement S1 of the Supplementary Materials.

### 6.2. Weight Assignment as a Design Variable

**Figure 5:**
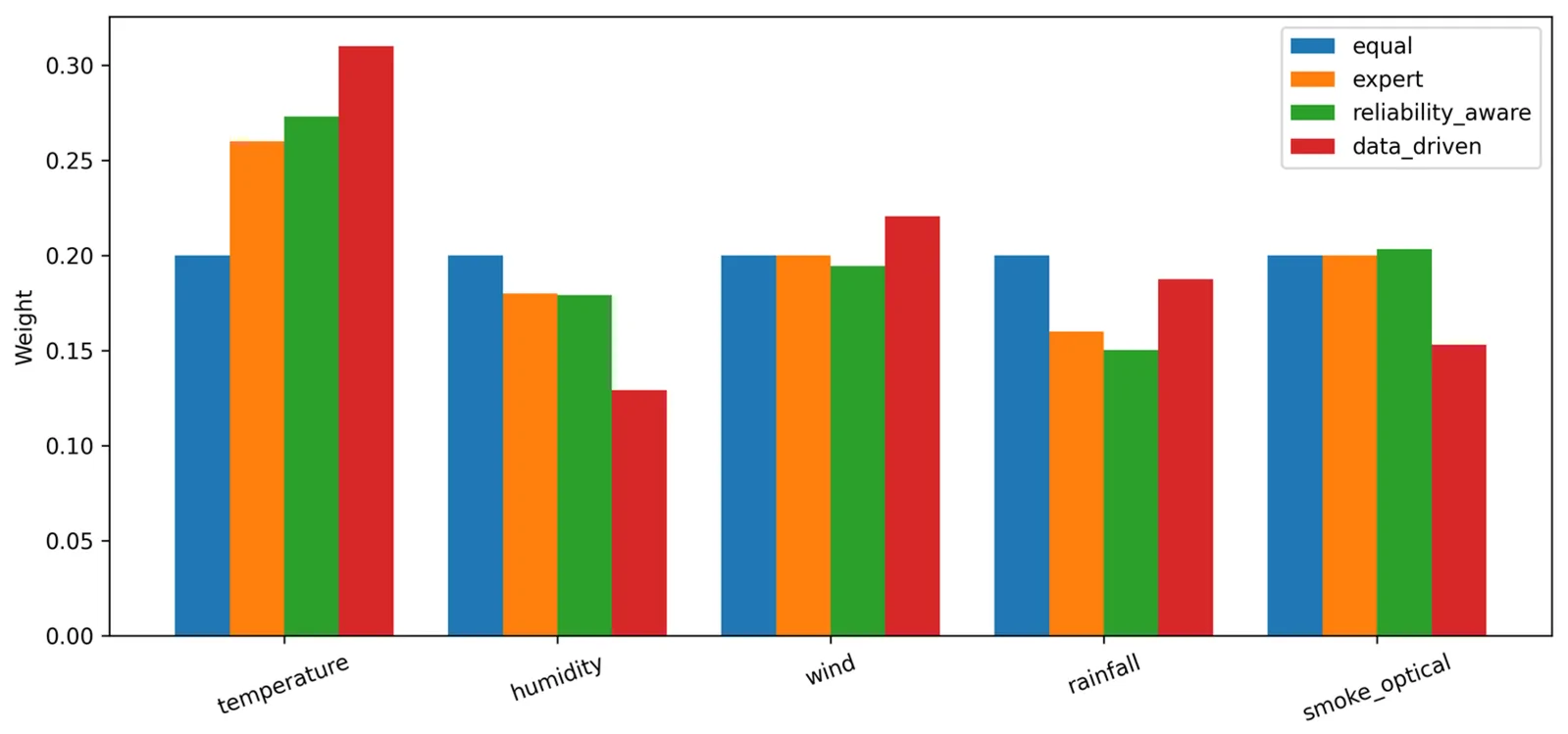
Comparison of weight-assignment strategies for event-level stochastic fusion.

The second major result is that weight assignment is a first-order design variable of the frontend rather than a secondary tuning detail. Using the same five-channel benchmark, four weighting strategies were compared: equal, expert, reliability-aware, and data-driven. A comparison of the tested weighting strategies is presented in Figure 5.

The figure summarizes the behavior of equal, expert, reliability-aware, and tested data-driven weighting, highlighting the effect of weight selection on inference accuracy and threshold consistency.

The comparison showed that naive equal weighting was consistently inferior to the better-structured alternatives, while reliability-aware weighting provided the best overall performance in the tested benchmark.

At N = 256, the stochastic estimate produced with equal weights yielded a MAE of 0.071738 relative to the reference event model, whereas expert weights reduced the MAE to 0.056903. Reliability-aware weighting improved it further to 0.053950, while the tested data-driven weighting variant reached 0.070957. The same ordering was observed in RMSE and threshold agreement. In particular, threshold agreement at N = 256 was 0.760667 for equal weighting, 0.833000 for expert weighting, 0.844667 for reliability-aware weighting, and 0.761000 for the tested data-driven variant. These results indicate that incorporating channel reliability into the weighting rule improves not only average error but also decision consistency.

This advantage remained visible at shorter stream lengths, which is especially relevant for resource-constrained stochastic inference. At N = 16, the MAE was 0.076646 for equal weighting, 0.064345 for expert weighting, and 0.062157 for reliability-aware weighting, again preserving the same ranking. Thus, the benefit of structured weighting was not limited to high-precision stochastic settings.

The robustness comparison under a noisy smoke-optical channel provided an additional perspective. Under this perturbation, reliability-aware weighting again produced the best results, with lower MAE and RMSE than equal, expert, or the tested data-driven weighting. This suggests that reliability-aware weighting is not only accurate under nominal conditions but also more stable under channel degradation.

The tested data-driven weighting strategy did not outperform the reliability-aware alternative in the present synthetic benchmark. This should be interpreted cautiously: the result reflects the specific benchmark setup and the particular calibration pipeline used here, not a general rejection of data-driven weighting. Nevertheless, it shows that a structured reliability-aware scheme can already provide strong practical performance without requiring a large-scale training pipeline. Full comparative tables and plots are reported in Supplement S2 of the Supplementary Materials.

Overall, the weighting study demonstrates that the frontend is not just a mapping layer. It is a controlled fusion architecture in which the semantics, trustworthiness, and relative importance of heterogeneous channels must be encoded explicitly. In the tested benchmark, reliability-aware weighting provided the best balance of accuracy, interpretability, and robustness.

### 6.3. Correlation and Synchronization Effects

**Figure 6:**
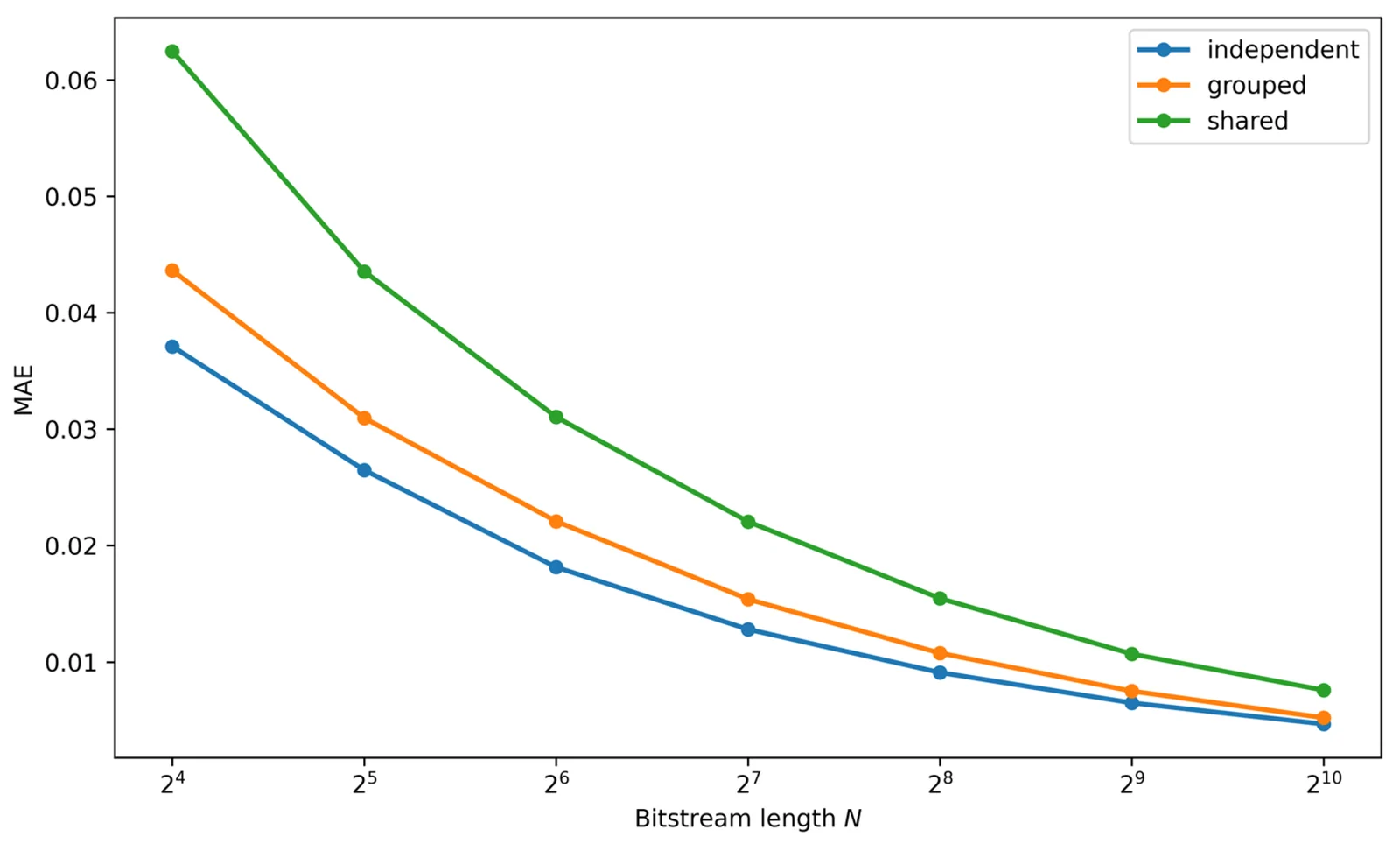
Effect of stochastic correlation policy on multisensor event inference.

The third major result concerns the role of stochastic correlation and synchronization. In the static stochastic fusion setting, the quality of event inference depended strongly on the inter-channel correlation structure of the Bernoulli streams, in agreement with the known sensitivity of stochastic computing to correlated bitstreams [1]. The effect of independent, grouped, and shared-randomness stream generation on inference quality is shown in Figure 6.

Mean absolute error (MAE) is shown as a function of bitstream length for independent, grouped, and shared-randomness stream generation under the perfectly synchronized condition.

Independent stream generation produced the best results, grouped generation produced intermediate results, and shared-randomness generation produced the worst results.

The ordering of the three policies reflects the role of stochastic correlation in bitstream-based inference. Independent generation reduces artificial inter-channel correlation and therefore allows the weighted fusion block to approximate the intended float-domain aggregation more faithfully. Grouped generation introduces partial correlation and therefore produces intermediate degradation. Shared-randomness generation creates the strongest correlation between channels, which distorts the effective stochastic representation and increases inference error. Thus, Figure 6 shows that randomness policy is not only an implementation detail but a frontend design variable.

At N = 256, the independent policy yielded MAE = 0.009094, RMSE = 0.011500, and threshold agreement = 0.960000. Under grouped correlation, the corresponding values were MAE = 0.010764, RMSE = 0.013485, and threshold agreement = 0.950333. Under shared randomness, performance degraded to MAE = 0.015462, RMSE = 0.019574, and threshold agreement = 0.927000. This ranking was preserved across the tested stream lengths. The effect was particularly pronounced at small N: at N = 16, shared randomness produced markedly higher error and lower threshold agreement than the independent policy. These results establish decorrelation as a system-level requirement for accurate stochastic multisensor fusion. Full static correlation results are given in Supplement S3 of the Supplementary Materials.

The static synchronization experiments, however, showed only negligible sensitivity to circular bitstream shifts. This was an expected limitation of the static formulation, because full-stream probability estimation depends mainly on empirical means, which are invariant under circular permutation. This observation motivated the introduction of the time-varying formulation.

**Figure 7:**
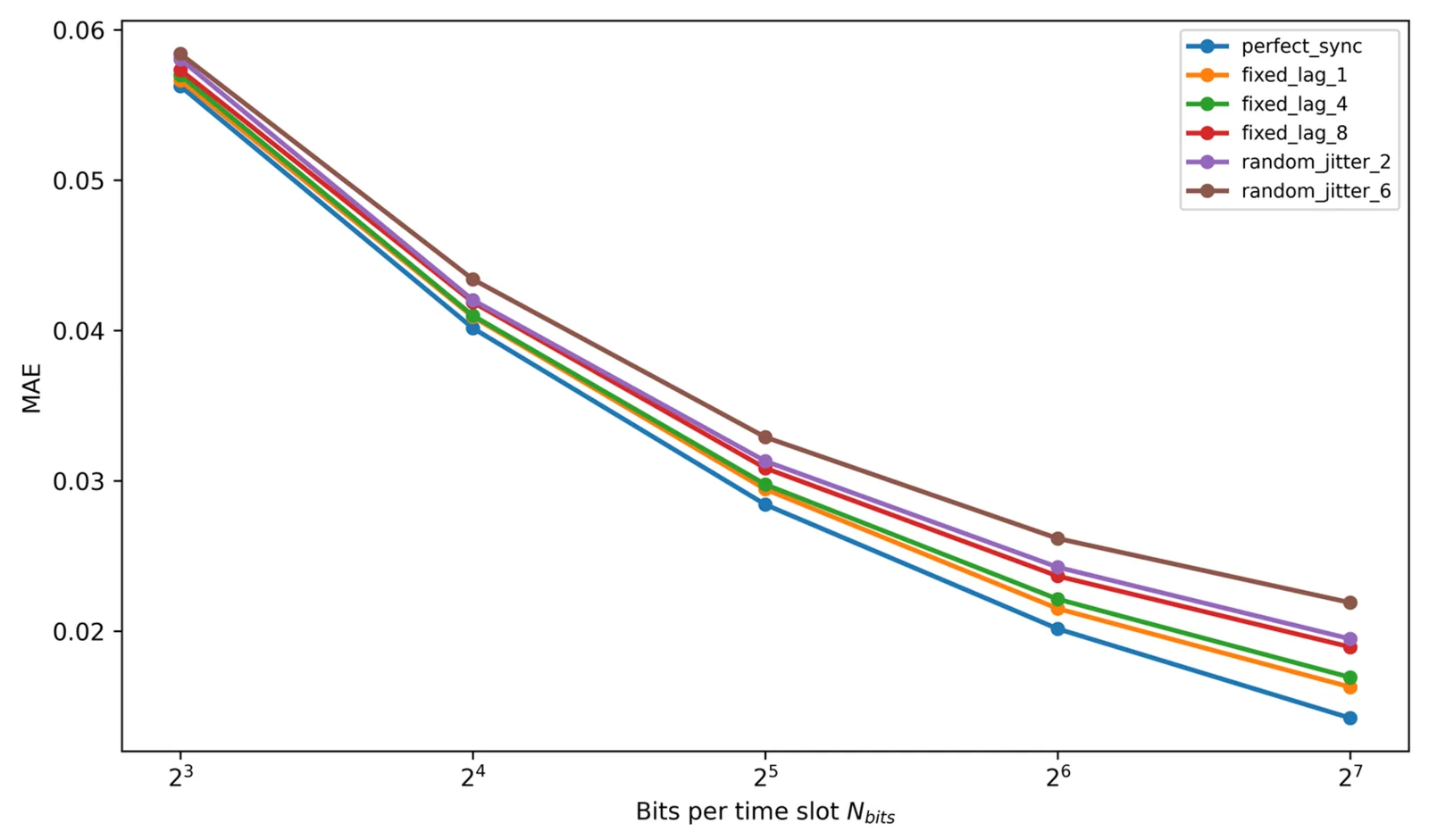
Time-varying synchronization effects on multisensor event inference.

In the time-varying slot-wise setting, synchronization became a measurable factor. Time-varying synchronization effects under lag and jitter conditions are reported in Figure 7.

Mean absolute error (MAE) is shown as a function of bits per time slot for the independent stochastic generation policy under perfect synchronization, fixed lag, and random jitter conditions.

Figure 7 extends the decorrelation analysis to time-varying event inference. In the static setting, some synchronization shifts can be partly hidden because full-stream averages remain similar. In the slot-wise formulation, however, fixed lag and random jitter directly distort the temporal alignment of channel evidence. The resulting increase in MAE and peak-alignment error indicates that synchronization is especially important when the target event evolves over time and when the frontend must track the event trajectory rather than only estimate an average event probability.

Once each channel was represented by a temporal probability trajectory *p*_*i*_(*t*) and event inference was performed slot by slot, temporal misalignment between channels degraded event tracking quality. Under independent stream generation, the best result was obtained in the perfectly synchronized case, with MAE = 0.020144, RMSE = 0.025274, threshold agreement = 0.985234, and peak alignment error = 11.9425. As the synchronization mismatch increased, performance degraded gradually. For example, under independent generation, MAE increased from 0.020144 in the perfect synchronization case to 0.023654 under fixed_lag_8.

The same qualitative behavior was observed for grouped generation, while shared-randomness policies remained inferior overall. In the time-varying benchmark, grouped perfect synchronization gave MAE = 0.025063, RMSE = 0.031457, and threshold agreement = 0.978398, whereas shared perfect synchronization yielded MAE = 0.037996, RMSE = 0.047630, and threshold agreement = 0.948730. Thus, both correlation structure and synchronization quality affected temporal event inference, with independent synchronized streams providing the best overall behavior.

The peak alignment error proved especially informative in the time-varying setting. It captured degradation in temporal event localization that was not always fully reflected by amplitude-based metrics alone. The metric decreased as the number of bits per time slot increased, but remained systematically worse for grouped and shared policies than for independent generation. This confirms that synchronization and decorrelation are not merely implementation details; they directly affect the frontend’s ability to track event dynamics in time.

Overall, the correlation and synchronization results support two distinct but complementary conclusions. First, in static stochastic fusion, inter-stream decorrelation is a first-order requirement. Second, in time-varying fusion, synchronization becomes an additional first-order factor, because temporal event inference is sensitive to slot misalignment. Detailed plots, heatmaps, and temporal tracking examples are provided in Supplements S3 and S3b of the Supplementary Materials.

### 6.4. Robustness Under Channel Degradation and Weight Mismatch

**Figure 8:**
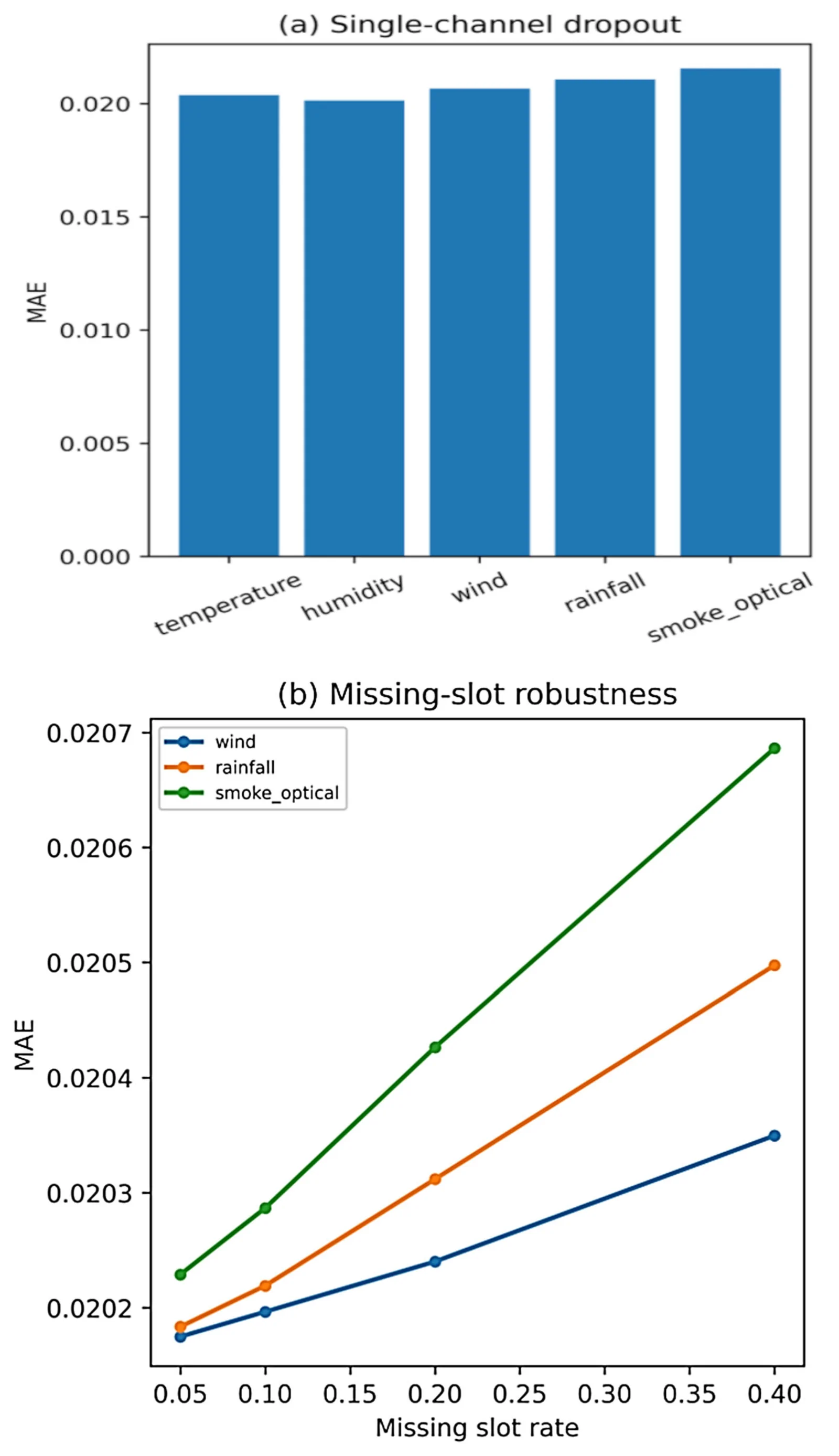

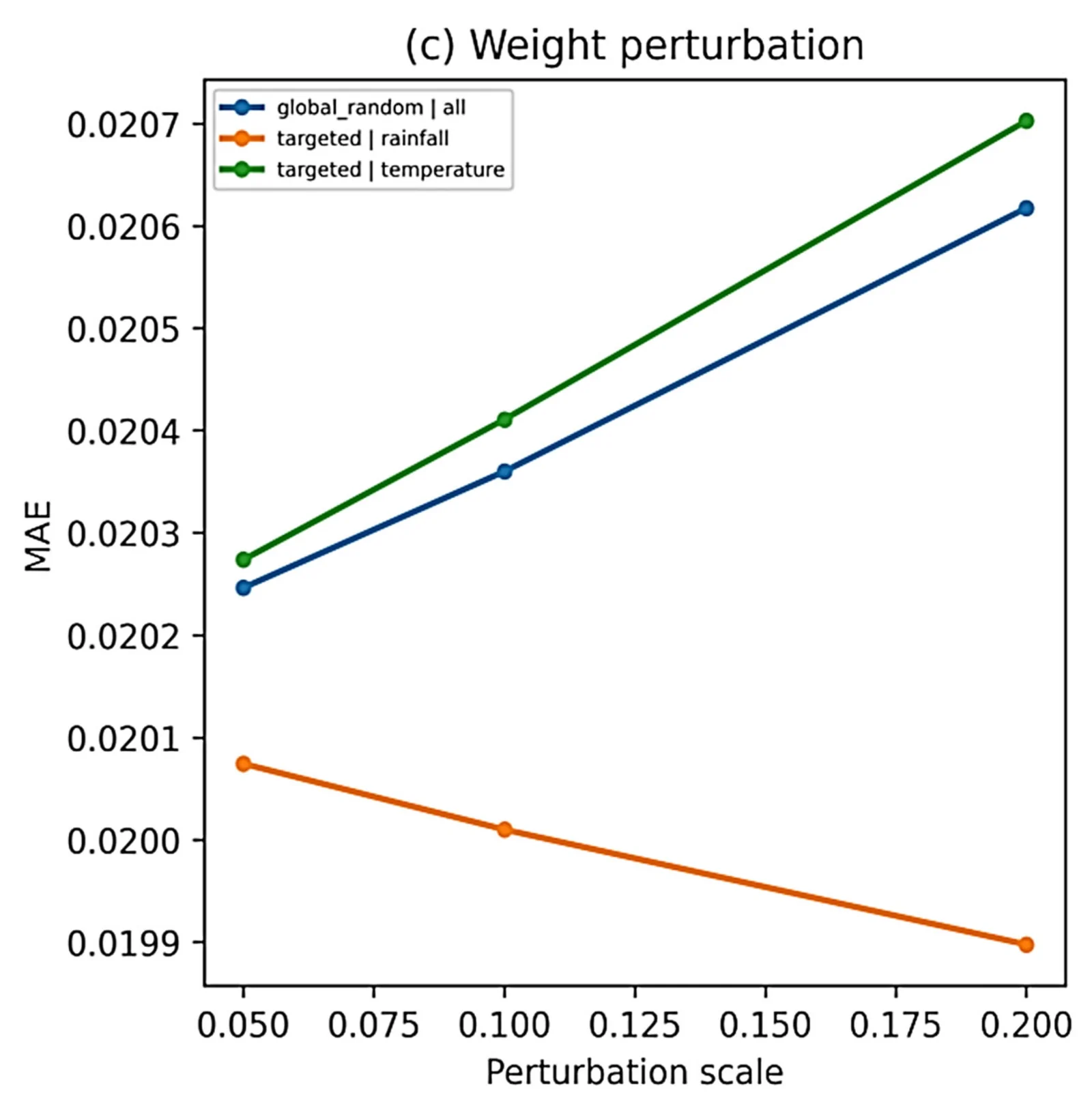
Robustness of the proposed frontend under representative degradation and misconfiguration scenarios. (**a**) Sensitivity to single-channel dropout. (**b**) Event-inference degradation under missing-slot corruption. (**c**) Sensitivity to weight perturbation under global and targeted mismatch conditions.

The robustness experiments showed that the proposed frontend degrades gracefully under several classes of perturbations, although different channels and design variables affect the system unequally. The robustness of the frontend under channel degradation and weight perturbation is summarized in Figure 8.

The behavior in Figure 8b,c should be interpreted as evidence of non-uniform channel criticality rather than as a failure of the common stochastic representation. Replacing a channel with a neutral value removes its event-specific evidence while preserving the formal input dimensionality of the fusion block. Therefore, channels carrying stronger event evidence, especially the smoke_optical channel in this benchmark, produce larger degradation when corrupted or removed. Missing-slot corruption has a similar interpretation: the frontend remains functional under partial data loss, but the magnitude of degradation depends on which channel is affected and how strongly that channel contributes to the event estimate. These results show that robustness must be evaluated jointly through average error, threshold agreement, and temporal tracking metrics.

This non-uniform behavior is itself an important architectural result, because it reveals which channels are critical and which perturbations are most damaging.

In the additive-noise study, the average amplitude error did not always increase monotonically with noise severity. In some cases, MAE decreased slightly as noise increased. For example, in the temperature-noise scenario, the baseline MAE of 0.020144 changed to 0.018756 at the highest tested noise level. Similar mild decreases were observed in the humidity and smoke-optical noise scenarios. However, this should not be interpreted as a genuine improvement in inference quality. The more decision-oriented metrics showed the opposite tendency: threshold agreement decreased and peak alignment error increased under stronger noise. For instance, with strong noise in the temperature channel, threshold agreement dropped from 0.985234 to 0.953164, while peak alignment error increased beyond 13 slots. This indicates that amplitude-only metrics can understate degradation when noise blurs decision boundaries or temporal peak localization.

The channel-dropout study provided a clearer picture of channel criticality. Among the five baseline channels, smoke_optical was the most critical: replacing it with a neutral value increased MAE to 0.021550, reduced threshold agreement to 0.926582, and increased peak alignment error to 17.99. Rainfall was the next most critical, whereas humidity had the smallest effect on overall performance. These results show that the frontend does not treat all channels equally in practice, even if they are all formally represented in the same stochastic format. Channel-dropout analysis, therefore, provides a meaningful way to identify dominant evidence sources in a heterogeneous event-inference system.

The missing-slot study further demonstrated graceful degradation. As the fraction of missing slots increased, MAE rose gradually rather than catastrophically. Again, smoke_optical was among the most sensitive channels, while wind was less sensitive. Thus, the system preserves functionality under partial data loss, but performance depends on which channel is degraded and how informative that channel is for the event.

Weight perturbation experiments revealed that errors in dominant channels are more harmful than errors in weaker ones. Targeted perturbation of the strongest informative channel produced greater degradation than comparable perturbation of a weaker channel. This result is consistent with the weighting study and reinforces the interpretation that the frontend is most sensitive to miscalibration, where informative channels carry a large share of the fusion burden.

Together, these robustness results support four points. First, the frontend is not fragile: it degrades progressively under realistic corruption scenarios. Second, channel criticality is non-uniform and can be quantified. Third, robustness must be assessed with several metrics simultaneously, because MAE alone may obscure threshold or temporal degradation. Fourth, dominant informative channels require the most careful calibration and protection. Full robustness tables and plots are reported in Supplement S4 of the Supplementary Materials.

### 6.5. Scaling Behavior Under Increasing Channel Count

**Figure 9:**
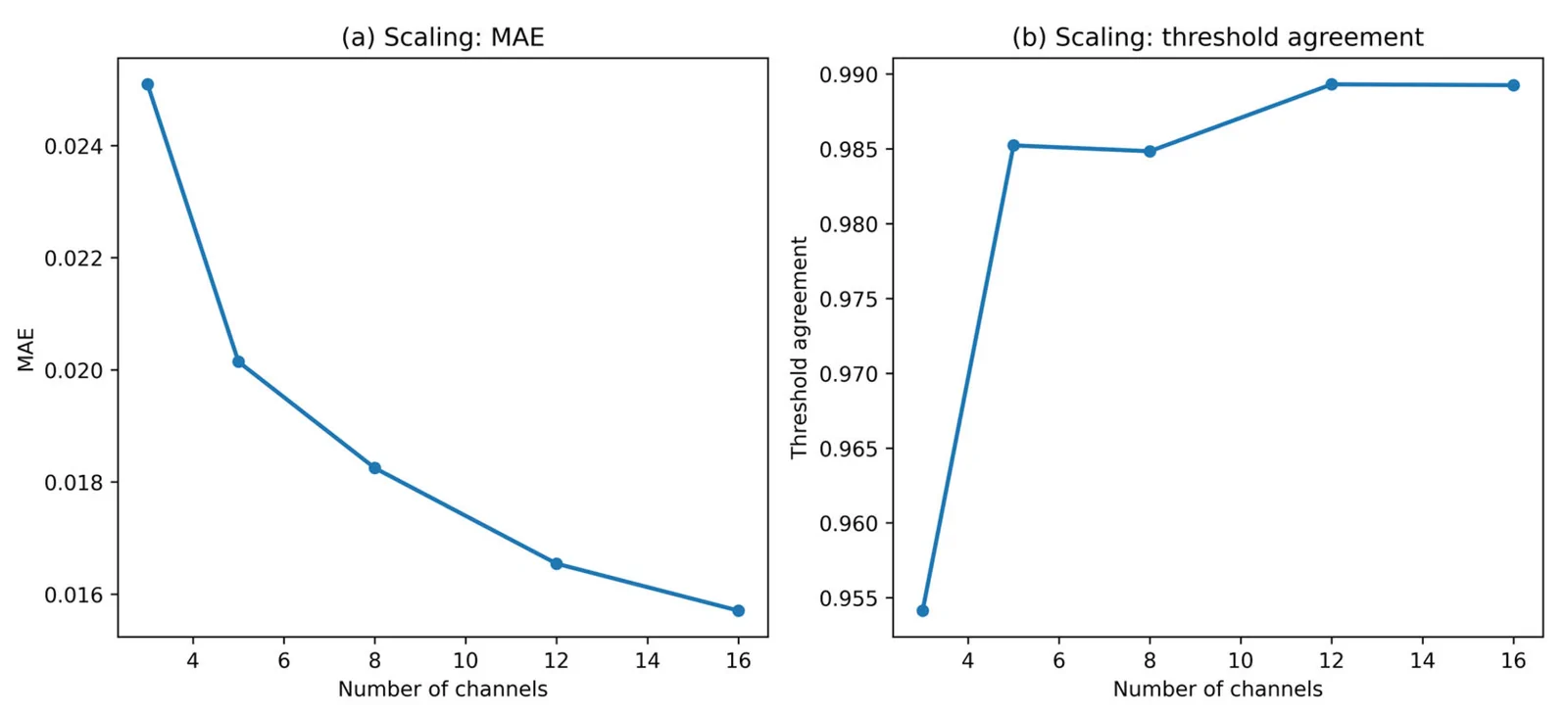
Scaling behavior of the event-oriented frontend with increasing heterogeneous channel count. (**a**) Mean absolute error (MAE) as a function of the number of channels. (**b**) Threshold agreement as a function of the number of channels. The results show favorable scaling in the tested benchmark despite the inclusion of mixed informative, weak, redundant, conflicting, and noisy channels.

The scaling study showed that, in the tested benchmark, increasing the number of channels from 3 to 16 improved event-inference quality despite the inclusion of weak, redundant, conflicting, and noisy channels. The effect of increasing channel count on event-inference quality is shown in Figure 9.

This result is important because it suggests that the proposed frontend can benefit from heterogeneous expansion, provided that the added channel set still contains sufficient useful structure.

With three channels, the frontend achieved MAE = 0.025091 and threshold agreement = 0.954141. Expanding to the five-channel baseline reduced MAE to 0.020144 and increased threshold agreement to 0.985234. Further expansion to 8, 12, and 16 channels reduced MAE to 0.018249, 0.016544, and 0.015707, respectively, while threshold agreement remained high and even increased slightly, reaching 0.989258 in the 16-channel case. Peak alignment error also improved from 11.9425 in the five-channel baseline to 10.5400 and 10.4525 in the 12- and 16-channel configurations.

This trend should be interpreted carefully. The result does not imply that adding channels always improves event inference in all systems. Rather, in the present benchmark, the added channels did not overwhelm the fusion process with useless or contradictory information; enough informative structure remained dominant that the additional evidence improved the aggregate event estimate. In other words, the scaling advantage depended on the mixture of channel types and on the frontend’s ability to absorb heterogeneous information through weighting and stochastic fusion.

This is an important practical conclusion. An architecture-level event-oriented frontend should not be evaluated only in minimal sensor configurations; it should also be assessed under realistic growth in channel count and diversity. The present results indicate that the proposed architecture scales favorably under the tested benchmark conditions, while also preserving robustness to mixed channel quality. Detailed scaling configurations and full metric tables are provided in Supplement S4 of the Supplementary Materials.

### 6.6. Photonic-Interface Robustness and Literature-Grounded Validation

The frontend evaluation includes two complementary validation layers beyond the baseline synthetic benchmark. Supplement S5 of the Supplementary Material characterizes first-order optical-interface nonidealities of the polarization-compatible layer, whereas Supplement S6 evaluates the same event-oriented stochastic pipeline using a literature-grounded 520 nm optical smoke-response channel.

In the photonic-interface analysis, the tested perturbations include differential loss, polarization leakage, detector gain imbalance, offsets, and additive noise. The results show that first-order optical nonidealities initially affect the soft-domain behavior of the interface by reducing analog H/V state separation and increasing soft-domain event error. In particular, leakage produces a clear degradation trend, reducing the analog H/V separation metric QSeparation and increasing the soft-domain event error MAE_soft. Hard bit errors remain small over the milder individual perturbation ranges but emerge under stronger combined degradation, indicating the onset of hard-decision failure. This supports the interpretation that soft-domain metrics are early indicators of optical-interface degradation, whereas BER becomes informative mainly in stronger degradation regimes.

The literature-grounded validation in Supplement S6 of the Supplementary Materials uses a digitized 520 nm smoke-layer transmittance curve reported in a published multi-wavelength densitometer study [20]. This case is therefore interpreted as an external-data-based validation layer, not as an independent experimental demonstration by the authors. The transmittance signal is converted into optical obscuration and then normalized as an event-oriented smoke-probability channel. This externally grounded optical channel is combined with controlled semi-real environmental proxy channels and processed through the same event-oriented mapping, Bernoulli bitstream generation, and float-versus-stochastic inference pipeline used in the main benchmark. At N = 256, the stochastic estimate remained close to the float reference, with MAE = 0.008003, RMSE = 0.010363, and threshold agreement = 0.973422. Robustness checks with optical-channel noise and missing optical samples showed controlled degradation relative to the unperturbed baseline while preserving stable stochastic tracking of the perturbed float reference. These results indicate that the proposed frontend can process an externally grounded optical smoke-response signal while preserving the same architecture-level stochastic inference pipeline.

### 6.7. Summary of Main Findings

Across all experimental stages, the results support a coherent architectural interpretation of the frontend. First, heterogeneous sensors can be mapped into a common event-oriented stochastic representation with controllable convergence to a float reference. Second, weight assignment materially affects fusion quality, with reliability-aware weighting emerging as the strongest tested strategy in the current benchmark. Third, decorrelation is essential for accurate stochastic multisensor fusion, and synchronization becomes essential once the event model is explicitly time-varying. Fourth, the frontend exhibits graceful degradation under noise, dropout, missing data, and weight perturbation, while remaining sensitive to channel criticality. Finally, scaling can improve inference quality when the added heterogeneous channels still preserve sufficient informative structure.

These findings collectively support the main thesis of this paper: the proposed architecture-level event-oriented frontend is not merely a signal-format converter, but a structured multisensor architecture whose performance is governed by event-oriented mapping, stochastic precision, weighting, decorrelation, synchronization, robustness, and scaling. Detailed computational evidence for each of these claims is provided in Supplements S1–S6 of the Supplementary Materials.

## 7. Discussion

The results support the interpretation of the proposed framework as an architecture-level event-oriented frontend rather than as a task-specific fusion script or a signal-representation module. The key architectural idea is that heterogeneous sensor channels do not need to be made identical in their raw physical units before entering a photonic stochastic system. Instead, they can be translated at the edge into normalized contributions to an event of interest, and only then fused in a common stochastic domain. This shift is important because it aligns the frontend with the actual inference objective: in many practical sensing systems, the desired output is not waveform reconstruction but estimation of a risk, event, anomaly, or decision state.

The first important implication of this study is that event-level unification is sufficient for meaningful multisensor interfacing. The baseline results showed that strongly heterogeneous channels can be mapped into a shared probability space and then encoded into Bernoulli bitstreams while preserving convergence toward a float reference model. This means that the frontend can act as a representation bridge between diverse sensor modalities and a downstream stochastic processor without requiring a conventional feature-space homogenization pipeline. In architectural terms, the frontend transforms heterogeneity from a raw-data incompatibility problem into a controlled event-probability mapping problem.

The second important implication is that weighting, decorrelation, and synchronization must be treated as primary design variables, not secondary implementation details. The weighting study showed that naive equal weighting is inadequate when channels differ in reliability and inferred relevance, and that a reliability-aware strategy can outperform both equal weighting and the tested data-driven calibration variant in the current benchmark. This suggests that even before introducing complex learning pipelines, practical multisensor systems can gain measurable benefit from explicitly modeling channel trustworthiness in the fusion rule. For a journal such as Sensors, this is an important systems-level message: the quality of event inference depends not only on sensor hardware and not only on the downstream classifier but also on how the interface interprets and weights each channel.

The decorrelation results reinforce a classical but often underemphasized property of stochastic computing: shared randomness is harmful when multiple channels are intended to contribute independent evidence. In the present study, independent stream generation consistently produced the best event-inference accuracy, grouped generation gave intermediate performance, and shared-randomness generation performed worst. This result is especially relevant because an architecture-level event-oriented frontend could otherwise appear to be only a semantic mapping layer rather than a complete stochastic inference interface. The experiments show that it is more than that: the frontend is also responsible for preserving statistical independence when the fusion logic requires it. In other words, the interface is not only about what probabilities are assigned to channels but also about how those probabilities are physically or algorithmically realized as stochastic streams.

The time-varying synchronization study adds another layer of practical relevance. In the static setting, synchronization offsets had little visible effect because full-stream averages are insensitive to circular reordering. However, once inference was reformulated as a time-resolved slot-wise process, synchronization became a measurable factor. This distinction is conceptually useful. It shows that synchronization should not be discussed in the abstract, but in relation to the temporal semantics of the target task. If the goal is only average event probability over a long window, synchronization may be less critical. If the goal is event tracking in time, then lag and jitter directly degrade performance. The present results, therefore, suggest that synchronization requirements depend on whether the stochastic frontend supports static decision support or dynamic event monitoring.

The robustness analysis provides an additional systems-level message: the frontend exhibits graceful degradation, but not all degradations are equivalent. Channel dropout, missing-slot corruption, and weight mismatch do not affect all channels equally. In the tested benchmark, the optical smoke-related channel emerged as one of the most critical evidence sources, while other channels were less decisive. This non-uniformity is not a weakness; rather, it is valuable diagnostic information. It means that robustness studies can be used not only to assess failure tolerance but also to identify the channels that deserve stronger protection, calibration, redundancy, or reliability-aware weighting in a real deployment. Similarly, the weight-perturbation study showed that errors in dominant informative channels are more harmful than comparable errors in weak channels, which is highly relevant for practical calibration workflows.

One of the more subtle findings of the robustness stage is that different metrics capture different failure modes. In particular, additive noise did not always increase MAE, yet it often reduced threshold agreement and worsened peak alignment. This indicates that average amplitude fidelity is not sufficient for evaluating event-level frontends. A system may appear stable under one metric while already losing reliability in thresholded decisions or temporal localization. For that reason, the joint use of MAE, RMSE, threshold agreement, and peak alignment error is not only methodologically justified but necessary. This point may also help position this paper within the sensor-systems literature, where average regression metrics alone are sometimes insufficient to capture operational decision quality.

The scaling results are encouraging, but they should be interpreted carefully. In the tested benchmark, increasing the number of channels improved performance even when the added set included weak, redundant, conflicting, and noisy channels. This suggests that the proposed frontend can exploit heterogeneous expansion effectively when useful structure remains dominant. However, this should not be overgeneralized into the claim that adding channels is always beneficial. In real systems, the value of scaling will depend on the ratio of informative to misleading inputs, on the quality of weighting, and on whether the frontend can suppress harmful correlations and misalignment. Thus, the present result is best viewed as evidence that the architecture can scale favorably under mixed-quality conditions, not as proof of unconditional monotonic benefit.

These findings also help clarify the intended scope of this paper relative to neighboring research directions. This work is not centered on compact stochastic encoding of a single waveform, harmonic factorization, or reconstruction fidelity. Those are valid topics, but they address a different problem. The contribution here is architectural: an architecture-level event-oriented stochastic frontend for heterogeneous sensors, designed for event inference and photonic compatibility. The frontend is evaluated not only by representational convergence but also by the system-level factors that determine whether such an interface is practically useful: weighting, decorrelation, synchronization, robustness, and scaling.

At the same time, several limitations should be stated clearly. The present study remains primarily architecture-oriented and does not report a complete fabricated photonic hardware prototype. The polarization-compatible optical interface is introduced as a physically meaningful interface layer, and Supplement S5 of the Supplementary Material evaluates its first-order nonidealities, including differential loss, leakage, detector imbalance, offsets, and noise. However, full device-level modeling of a specific modulator, detector, material stack, bandwidth limit, thermal behavior, or phonon-related mechanism remains outside the scope of the present work.

The benchmark scenarios are controlled and synthetic by design because their purpose is to isolate frontend design variables such as weighting, decorrelation, synchronization, robustness, and scaling. To reduce the gap between the controlled benchmark and externally grounded sensing behavior, Supplement S6 provides a semi-real validation case based on a digitized 520 nm optical smoke-transmittance curve. This does not constitute a new independent field experiment by the authors, but it demonstrates that the same event-oriented stochastic frontend can process a literature-grounded optical sensing channel within the proposed architecture.

Finally, the tested data-driven weighting strategy should be interpreted as one simple calibration variant. It does not exclude stronger Bayesian, neural, or context-adaptive weighting methods, which may provide improved performance in future application-specific datasets.

These limitations point naturally to future work. A subsequent hardware-oriented study should instantiate the proposed interface in a concrete polarization-based photonic pipeline using selected electro-optic modulators, polarization-maintaining routing elements, PBS/analyzer stages, and calibrated photodetectors, followed by experimental characterization of extinction ratio, crosstalk, detector noise, bandwidth, timing jitter, and thermal drift. Another direction would be to validate the frontend on real heterogeneous sensing datasets, especially in event-detection domains where channel reliability varies over time. Context-adaptive weighting, online recalibration, and joint optimization of edge mapping with downstream stochastic fusion are also promising extensions. More broadly, the present results suggest that the proposed frontend can serve as a design template for photonic edge-inference systems in which heterogeneous sensing must be integrated through a common probabilistic interface rather than through raw signal transport.

Overall, the discussion supports a clear conclusion: the proposed architecture is meaningful precisely because it treats the sensor interface as an inference-aware stochastic system. By shifting the edge representation from raw heterogeneous measurements to unified event-oriented stochastic contributions, it creates a practical pathway between multisensor acquisition and future photonic stochastic processors.

## 8. Conclusions

This paper proposed an architecture-level event-oriented stochastic frontend for heterogeneous sensors in photonic stochastic systems. The central idea was to convert physically different sensor measurements into a common event-oriented probability space and then represent these contributions as Bernoulli bitstreams compatible with a polarization-based optical interface. In this way, the frontend serves as a bridge between heterogeneous sensing and stochastic photonic processing without requiring direct fusion in raw physical units.

The results showed that the proposed frontend provides controlled convergence from stochastic event inference to a float reference model as the bitstream length increases. This confirms that heterogeneous channels can be unified into a common stochastic event representation while preserving useful inference fidelity.

This study also demonstrated that the weighting strategy is a key design variable of the frontend. In the tested benchmark, reliability-aware weighting provided the best overall balance of accuracy, threshold consistency, and robustness, outperforming naive equal weighting and the tested data-driven weighting variant.

A further important conclusion is that stochastic stream decorrelation and temporal synchronization must be treated as core architectural constraints. Independent bitstream generation consistently produced better event-inference quality than grouped or shared-randomness policies, while synchronization mismatch became clearly measurable in the time-varying formulation, where it degraded temporal event tracking.

The robustness and scaling studies showed that the frontend degrades gracefully under channel corruption, missing data, and weight perturbation, while also exhibiting non-uniform channel criticality. In the tested heterogeneous benchmark, scaling from smaller to larger channel sets improved inference quality, indicating that the proposed architecture can benefit from structured multisensor expansion when informative contributions remain dominant.

The validation results support the practical interpretation of the proposed frontend architecture. First-order photonic-interface nonidealities mainly reduce analog state separation and increase soft-domain event error before causing hard-decision degradation under stronger combined perturbations. The literature-grounded smoke-response case further demonstrates that the same event-oriented stochastic pipeline can process an optical channel derived from experimentally reported smoke-transmittance behavior. Together, these results reinforce the role of the proposed frontend as an architecture-level bridge between heterogeneous sensing and photonic stochastic processing.

Overall, the presented framework establishes a practical event-level sensor-to-stochastic interface for photonic systems. The results suggest that heterogeneous sensing can be coupled to future photonic stochastic processors through a unified frontend in which weighting, decorrelation, synchronization, robustness, and scaling are treated as first-order system design variables.

## Data Availability

All data supporting the reported results are included in the article and in the Supplementary Material. The Supplementary Material contains the reproducible computational notebooks, scripts, generated result files, and supporting figures and tables used in the present study. The main benchmark results are based on computationally generated controlled scenarios prepared specifically for this work. The semi-real optical smoke-response validation uses a digitized 520 nm smoke-layer transmittance curve derived from externally published experimental data, as described in Supplement S6. A preprint version of this work is available in Preprints [21].

## Supplementary Materials

The following supporting information can be downloaded at: https://www.mdpi.com/article/10.3390/s26175565/s1: Supplement S1: Baseline frontend validation and stochastic convergence analysis; Supplement S2: Weighting-strategy comparison for event-level fusion; Supplement S3: Static stochastic-stream decorrelation analysis; Supplement S3b: Time-varying synchronization and temporal event-tracking analysis; Supplement S4: Robustness and scaling analysis under channel degradation, missing data, weight perturbation, and increasing channel count; Supplement S5: First-order photonic-interface nonideality analysis of the polarization-compatible H/V optical binary-state layer; Supplement S6: Semi-real literature-grounded optical smoke-response validation based on a digitized 520 nm smoke-layer transmittance curve. The supplementary package also includes reproducible computational notebooks, scripts, generated result files, supporting figures, and tables.

## Abbreviations

The following abbreviations are used in this manuscript:

MAE	Mean absolute error
RMSE	Root mean square error
SC	Stochastic computing
P−bit	Probabilistic bit
H/V	Horizontal/vertical polarization states
CSV	Comma-separated values
JSON	JavaScript object notation
DOI	Digital object identifier
P(E)	Float reference event probability
$\hat{P}(E)\;\;\;$	Stochastic estimate of event probability
P(E,t)	Time-varying reference event probability
$\hat{P}(E,t)\;$	Time-varying stochastic estimate of event probability
$p_{i}\;$	Event-oriented probability contribution of sensor channel i
${\hat{p}}_{i}\;$	Bitstream-based estimate of channel probability
$p_{i}(t)$	Time-varying event-oriented probability contribution of channel i
${\hat{p}}_{i}(t)\;$	Slot-wise estimate of channel probability
$S_{i}\;$	Bernoulli bitstream generated from channel probability $p_{i}$
$S_{i}(t)\;$	Time-varying Bernoulli bitstream
$w_{i}\;$	Fusion weight of sensor channel i
θ	Decision threshold
α	Sigmoid slope parameter
N	Bitstream length
$N_{bits}\;$	Bits per time slot
T	Number of time slots
